# TCR catch bonds nonlinearly control CD8 cooperation to shape T cell specificity

**DOI:** 10.1038/s41422-025-01077-9

**Published:** 2025-02-27

**Authors:** Rui Qin, Yong Zhang, Jiawei Shi, Peng Wu, Chenyi An, Zhenhai Li, Nuo Liu, Ziyan Wan, Ting Hua, Xiaolong Li, Jizhong Lou, Weiwei Yin, Wei Chen

**Affiliations:** 1 Department of Cardiology of the Second Affiliated Hospital and Department of Cell Biology, Zhejiang University School of Medicine, Liangzhu Laboratory, Zhejiang University, Hangzhou, Zhejiang China; 2 State Key Laboratory of Epigenetic Regulation and Intervention, CAS Center for Excellence in Biomacromolecules, Institute of Biophysics, Chinese Academy of Sciences, Beijing, China; 3 University of Chinese Academy of Sciences, Beijing, China; 4 Key Laboratory for Biomedical Engineering of the Ministry of Education, and Zhejiang Provincial Key Laboratory of Cardio-Cerebral Vascular Detection Technology and Medicinal Effectiveness Appraisal, and College of Biomedical Engineering and Instrument Science, Zhejiang University, Hangzhou, Zhejiang China; 5 Tianjian Laboratory of Advanced Biomedical Sciences, Academy of Medical Sciences, Zhengzhou University, Zhengzhou, Henan China; 6 School of Biology and Engineering, Guizhou Medical University, Guiyang, Guizhou China; 7 Shanghai Key Laboratory of Mechanics in Energy Engineering, Shanghai Institute of Applied Mathematics and Mechanics, School of Mechanics and Engineering Science, Shanghai University, Shanghai, China; 8 Department of Hematology, The First Affiliated Hospital of USTC, Division of Life Sciences and Medicine, University of Science and Technology of China, Hefei, Anhui China; 9 National Key Laboratory of Immune Response and Immunotherapy & MOE Key Laboratory for Cellular Dynamics, School of Life Sciences, Division of Life Sciences and Medicine, University of Science and Technology of China, Hefei, Anhui China; 10 State Key Laboratory of Transvascular Implantation Devices, Hangzhou, Zhejiang China; 11 MOE Frontier Science Center for Brain Science and Brain-machine Integration, Zhejiang University, Hangzhou, Zhejiang China

**Keywords:** Single-molecule biophysics, Tumour immunology, Molecular modelling

## Abstract

Naturally evolved T-cell receptors (TCRs) exhibit remarkably high specificity in discriminating non-self antigens from self-antigens under dynamic biomechanical modulation. In contrast, engineered high-affinity TCRs often lose this specificity, leading to cross-reactivity with self-antigens and off-target toxicity. The underlying mechanism for this difference remains unclear. Our study reveals that natural TCRs exploit mechanical force to form optimal catch bonds with their cognate antigens. This process relies on a mechanically flexible TCR–pMHC binding interface, which enables force-enhanced CD8 coreceptor binding to MHC-α_1_α_2_ domains through sequential conformational changes induced by force in both the MHC and CD8. Conversely, engineered high-affinity TCRs create rigid, tightly bound interfaces with cognate pMHCs of their parental TCRs. This rigidity prevents the force-induced conformational changes necessary for optimal catch-bond formation. Paradoxically, these high-affinity TCRs can form moderate catch bonds with non-stimulatory pMHCs of their parental TCRs, leading to off-target cross-reactivity and reduced specificity. We have also developed comprehensive force-dependent TCR–pMHC kinetics-function maps capable of distinguishing functional and non-functional TCR–pMHC pairs and identifying toxic, cross-reactive TCRs. These findings elucidate the mechano-chemical basis of the specificity of natural TCRs and highlight the critical role of CD8 in targeting cognate antigens. This work provides valuable insights for engineering TCRs with enhanced specificity and potency against non-self antigens, particularly for applications in cancer immunotherapy and infectious disease treatment, while minimizing the risk of self-antigen cross-reactivity.

## Introduction

T-cell receptors (TCRs) recognize cognate peptides presented by major histocompatibility complexes (pMHCs), playing a central role in adaptive immunity by defending against pathogens and tumor cells.^1–3^ This recognition, aided by coreceptors such as CD8, must be highly specific to ensure effective immune surveillance and prevent autoimmunity.^4–8^ However, pathogens and tumor cells can often evade the immune surveillance through mutations in the antigens that weaken TCR–pMHC binding.^9–11^ To counteract this evasion, strategies have been developed to enhance TCR binding affinity. Yet, these approaches frequently result in off-target interactions with self-antigens, leading to tissue damage and limiting the success of TCR-based T-cell therapies.^12–18^ Understanding the molecular mechanisms underlying the specificity of naturally evolved TCRs is essential to address these challenges. It is also crucial to investigate how CD8 influences off-target cross-reactivity in engineered high-affinity TCRs and to elucidate the mechano-chemical basis governing TCR–pMHC interactions that dictate specificity and prevent off-target effects for improving the safety and efficacy of TCR-based immunotherapies.

Traditionally, TCR specificity and function were thought to depend on three-dimensional (3D) binding affinity, measured through methods like surface plasmon resonance (SPR). Higher TCR–pMHC affinity was assumed to improve specificity and activation.^19,20^ However, studies have shown that 3D affinity does not always correlate with TCR specificity and functional effectiveness.^21–23^ For instance, a high-affinity mutant of 2C-TCR (m33-TCR) was found to be less effective than its parental counterpart in slowing tumor growth in mice.^24^ These findings suggest that 3D affinity measurements may not accurately reflect the dynamics of TCR–pMHC interactions that occur at the two-dimensional (2D) interface where T cells engage antigen-presenting cells (APCs), often with assistance from coreceptors such as CD8. Previous studies have often relied on pMHC-tetramer staining to measure binding kinetics of pMHCs with TCRs expressed on T cells, but this method is prone to yield high false-positive rates for T cell activation, raising concerns about its reliability.^23^ Consequently, 2D binding assays have emerged as more physiologically relevant approaches for characterizing TCR–pMHC interactions. These assays better capture the conditions under which T cells interact with APCs, providing insights into the true specificity and functional effectiveness of TCRs in a biologically meaningful context.

While measurements of 2D binding affinity better correlate with TCR potency, they remain inadequate in distinguishing highly specific TCRs from those prone to off-target cross-reactivity.^22,25–27^ This limitation may stem from the exclusion of CD8 in many 2D assays, despite evidence indicating that CD8 influences TCR binding by reducing dissociation rates or prolonging bond lifetimes.^28,29^ Prolonged TCR–pMHC bond lifetimes are generally associated with enhanced T cell activation and TCR specificity.^6^ However, the “focusing hypothesis” posits that an optimal, rather than maximal, dwell-time is crucial for regulating T cell activation and TCR specificity.^5,30,31^ Extended bond lifetimes may, in some cases, inhibit T cell activation, as proposed by the “serial triggering model”.^19,32,33^ The strength of CD8–MHC binding also modulates TCR–pMHC dissociation and ligand recognition, further underscoring CD8’s selective role in shaping TCR specificity.^34,35^ Furthermore, the precise mechanism by which TCR–pMHC interactions coordinate with CD8 to regulate TCR specificity remains unclear. Key questions include whether CD8 selectively assists TCR binding to certain antigens, how TCR–pMHC interactions influence CD8 cooperation, and whether CD8 contributes to the cross-reactivity of engineered high-affinity TCRs.

Recent studies have underscored the critical role of mechanical forces in TCR antigen recognition.^1,2,36–43^ In the process of antigen scanning, T cells generate dynamic traction and tensile forces on TCR–pMHC complexes,^44,45^ which can induce conformational changes to promote the formation of catch bonds that stabilize TCRs' interactions with agonistic pMHCs.^23,27^ Unlike slip bonds, which weaken and shorten bond lifetimes under force, catch bonds strengthen TCR–pMHC interactions under force, making them uniquely suited to ensure TCR specificity.^23,27^ However, both naturally evolved TCRs with high specificity and engineered high-affinity TCRs with off-target cross-reactivity can form catch bonds with the same antigens,^26^ complicating efforts to distinguish between them.

In this study, we demonstrate that the strength of TCR catch bonds significantly influences CD8’s role in determining TCR specificity. Using single-molecule biophysical and functional analyses, we reveal that naturally evolved TCRs form optimal catch bonds with cognate pMHCs. These optimal bonds facilitate CD8 engagement to enhance TCR specificity by enabling sequential, force-induced conformational changes in TCR, pMHC, and CD8 molecules. In contrast, engineered high-affinity TCRs form overly tight binding interfaces with cognate pMHCs, disrupting the necessary force-induced conformational changes and impairing proper CD8 interaction with pMHCs. Paradoxically, these high-affinity TCRs can also establish moderate-strength catch bonds with non-stimulatory or self pMHCs, leading to off-target cross-reactivity, diminished TCR specificity, and reduced therapeutic potential. Our findings highlight the importance of balancing TCR catch-bond strength to optimize CD8-mediated TCR specificity while minimizing off-target cross-reactivity, offering critical insights for designing effective TCR-based cell therapies that can achieve enhanced potency and safety.

## Results

### Affinity enhancement of 2C-TCR attenuates T cell activation, antigen sensitivity and specificity

**Fig. 1:**
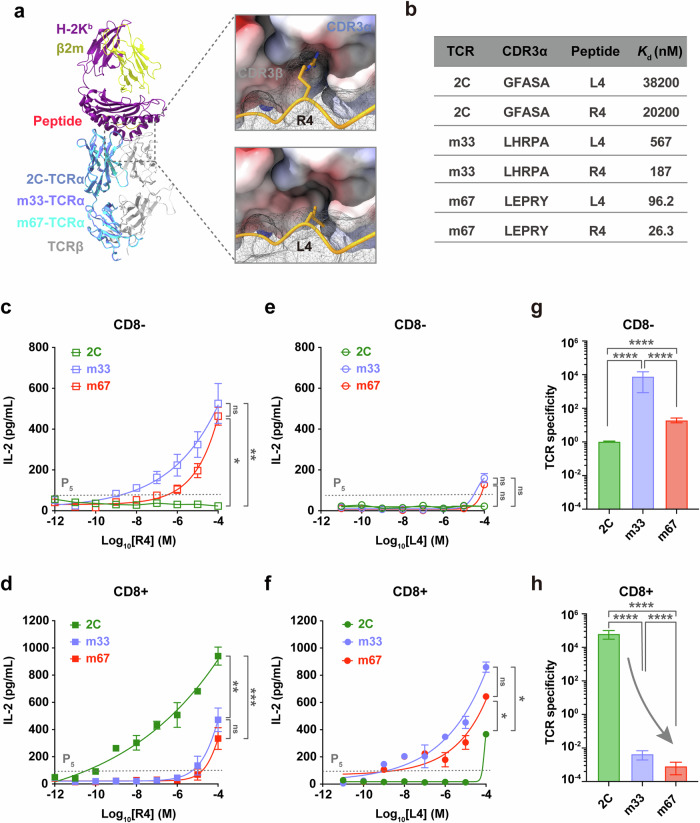
Strengthening TCR–pMHC 3D binding affinity by TCR CDR3α mutagenesis impairs the TCR specificity in the presence of CD8. **a** Structural overview of 2C-TCR (cornflower blue), m33-TCR (medium purple), and m67-TCR (cyan) in complex with R4-MHC are depicted. The R4 (SIYRYYGL) peptide, H-2K^b^ and β2-microglobulin within R4–MHC complex are highlighted in gold, purple, and yellow, respectively. A zoomed-in view of the interactions between the R4 peptide (SIYRYYGL, upper panel) or the L4 peptide (SIYLYYGL, bottom panel) and CDR3s of 2C-TCR is shown in dashed boxes on the left; the peptide is represented in yellow ribbon with the hotspot residue shown in stick, and CDR3s are represented in surface colored according to electrostatic properties (positive charge in red and negative charge in blue) on the right. **b** 3D binding affinity $({K}_{{{{\rm{d}}}}})$ of 2C-, m33-, and m67-TCRs binding to R4- or L4-MHCs, respectively. **c**–**f** IL-2 production of 2C, m33 and m67 hybridoma T cells expressing CD8 or not, when stimulated by RMA-S cells pulsed with different concentrations of R4 (**c**, **d**) or L4 (**e**, **f**) peptide. The data for both the presence and absence of CD8 were obtained from the same experimental batch. The TCR sensitivity is determined by the lowest antigen concentration that started to elicit the IL-2 production from hybridoma T cells (5% potency, or P_5_). The presented data represent one of three independent experiments. The analyses were conducted using the Mann–Whitney *U*-test. **g**, **h** The comparison of TCR specificity of 2C-, m33-, or m67-TCRs in the absence (**g**) or presence (**h**) of CD8. Error bars are ± SEMs. The analyses were conducted using unpaired student's *t*-tests. Statistical significance was indicated as follows: ns not significant, *P < 0.05, **P < 0.01, ***P < 0.001, ****P < 0.0001.

Studies have shown that the high-affinity variant (m33-TCR) was less potent in delaying tumor growth than its parental natural 2C-TCR in mice,^24^ prompting us to investigate how the 3D binding affinity $({K}_{{{{\rm{d}}}}})$ of the TCR–pMHC bi-molecular interaction can influence TCR specificity and off-target cross-reactivity. 2C-TCR specifically recognizes the SIY**R**YYGL peptide (R4) presented by H-2K^b^ (R4-MHC), where the critical residue at position 4 (arginine) plays a vital role in initiating and determining 2C-TCR antigen recognition via its interaction with 2C-TCR-CDR3β^27,46^ to stabilize the 2C-TCR–R4-MHC complex (Fig. 1a). Mutation of this residue to leucine alters the peptide sequence to SIY**L**YYGL (L4), significantly reducing the TCR–pMHC 3D binding affinity and rendering the peptide non-stimulatory for the 2C-TCR (Fig. 1b). Additionally, the binding strength is influenced by interactions between the TCR’s complementarity-determining regions (CDRs), particularly CDR3α motif, and the α_1_α_2_ domains of pMHC (Fig. 1b).^20^ Therefore, we engineered two TCR variants by mutating the essential GFASA motif within 2C-TCR-CDR3α to LHRPA or LERPY, generating the m33- or m67-TCRs, respectively.^20^ These engineered TCRs were then individually expressed, with or without CD8, on 58α-β- hybridoma T cells (Supplementary information, Fig. S1a, b) to investigate the dynamic molecular mechanism by which CD8 and TCR–pMHC binding cooperatively regulate TCR specificity and off-target cross-reactivity.

We examined the effector function (e.g., IL-2 releases) of T cells expressing 2C-, m33- or m67- TCRs in response to stimulation by the R4 or L4 peptides presented by RMA-S APCs in the presence or absence of CD8 (Fig. 1c–f; Supplementary information, Fig. S1c, d). The R4 peptide strongly stimulated 2C hybridoma T cells to produce substantial amounts of IL-2 when CD8 was present. IL-2 production was over 100-fold higher with CD8 than without, even at very low antigen concentrations (i.e., 10^–11^ M) (Fig. 1c, d; Supplementary information, Fig. S1c). Furthermore, the R4 peptide also stimulated m33 or m67 hybridoma T cells to release IL-2 at very low concentrations in the absence of CD8 (i.e., 10^–10^ M and 10^–8^ M) (Fig. 1c). However, when CD8 was present, the IL-2 production of m33 and m67 hybridoma T cells stimulated by R4 peptide did not significantly increase compared to conditions without CD8, across a wide range of antigen concentrations (i.e., from 10^–10^ to 10^–6^ M) (Fig. 1d; Supplementary information, Fig. S1c). Conversely, the L4 peptide failed to activate 2C hybridoma T cells, regardless of CD8 presence (Fig. 1e, f; Supplementary information, Fig. S1d), but was able to stimulate IL-2 release from m33 or m67 hybridoma T cells when CD8 was present, even at low antigen concentrations (i.e., 10^–9^ M for m33 hybridoma T cells and 10^–10^ M for m67 hybridoma T cells) (Fig. 1e, f; Supplementary information, Fig. S1d).

We subsequently compared the sensitivities and specificities of these three TCRs in recognizing different antigens. The sensitivity of each TCR–pMHC pair was calculated as the reciprocal of the lowest antigen concentration required to elicit IL-2 production by T cells (~5% of the maximum IL-2 production) within the measurable peptide concentration range (i.e., from 10^–12^ to 10^–4^ M) (Supplementary information, Fig. S1e, f). TCR specificity, representing the antigen discriminative power of TCRs, was shown as the ratio of TCR sensitivities for recognizing the cognate antigen (e.g., R4-MHC for 2C-TCR) over a non-stimulatory antigen (e.g., L4-MHC for 2C-TCR) (Fig. 1g, h). Our analysis revealed that these two parameters can be differentially regulated by the 3D affinity of TCR–pMHC binding and the presence of CD8. In the absence of CD8, enhancing 3D affinity led to the best TCR sensitivity and specificity for the m33-TCR among the three TCRs (Fig. 1g; Supplementary information, Fig. S1e, f). However, these enhancements were reduced when 3D affinity was further increased for the m67-TCR (Fig. 1g; Supplementary information, Fig. S1e, f). Surprisingly, when CD8 was present, our data showed that for R4-MHC, stronger 3D binding affinity with TCRs led to reduced TCR sensitivity and specificity (Fig. 1h; Supplementary information, Fig. S1e). Conversely, strengthening the 3D binding affinities of L4-MHC with the three TCRs resulted in increased TCR sensitivity (Supplementary information, Fig. S1f). Collectively, our results reveal that, within the 2C-TCR system, TCR sensitivity and specificity are enhanced by high-affinity mutation in TCR-CDR3α in the absence of CD8; but this effect is reversed when CD8 is present, suggesting distinct synergistic effects of CD8.

### In situ 2D TCR–pMHC binding affinity does not well reflect TCR specificity

Considering the physical restriction imposed by the 2D plasma membrane on TCR binding with pMHC,^22,47^ we questioned whether in situ 2D binding affinity could better correlate with TCR specificity. To address this, we characterized the 2D effective binding affinities of the three TCRs individually interacting with either R4- or L4-MHC using a single-cell adhesion frequency assay. Our results revealed an ~44-fold difference in the 2D effective binding affinities $({A}_{{{{\rm{c}}}}}{K}_{{{{\rm{a}}}}})$ between 2C-TCR interacting with R4-MHC (~0.16 × 10^–2^ μm^4^) and that interacting with L4-MHC (~0.37 × 10^–4^ μm^4^) (Supplementary information, Fig. S2 and Table S1). This aligns with the functional potencies of R4 and L4 in stimulating 2C hybridoma T cells. For m33-TCR, the 2D effective binding affinities for R4- was ~0.13 × 10^–2^ μm^4^, which is similar to that of 2C-TCR binding to R4-MHC, but showing no difference from the affinity of m33-TCR binding to L4-MHCs (~0.089 × 10^–2^ μm^4^) (Supplementary information, Fig. S2 and Table S1). Interestingly, m67-TCR also exhibited similar 2D effective binding affinities for R4-MHC (~0.16 × 10^–2^ μm^4^) and L4-MHC (~0.99 × 10^–3^ μm^4^). This result contradicts the antigen potencies in stimulating IL-2 release in vitro from corresponding TCR-expressing T cells (Supplementary information, Fig. S2 and Table S1). Furthermore, the Pearson correlation coefficient between the specificities of 2C-, m33-, and m67-TCRs and their corresponding ratios of 2D effective binding affinity for interacting with R4- over L4-MHC is −0.50 (Supplementary information, Fig. S4a, c and Table S7). This weak correlation suggests that relying solely on 2D TCR–pMHC binding affinity to predict TCR specificity is insufficient.

### The force-dependent TCR–pMHC bond lifetimes in the absence of CD8 also correlate poorly with TCR specificity

TCR–pMHC catch bonds have been reported as crucial for T cell activation.^23,27,48–50^ We further investigated whether, in the absence of CD8, force-dependent TCR–pMHC bond lifetimes could better correlate with TCR specificity.

**Fig. 2:**
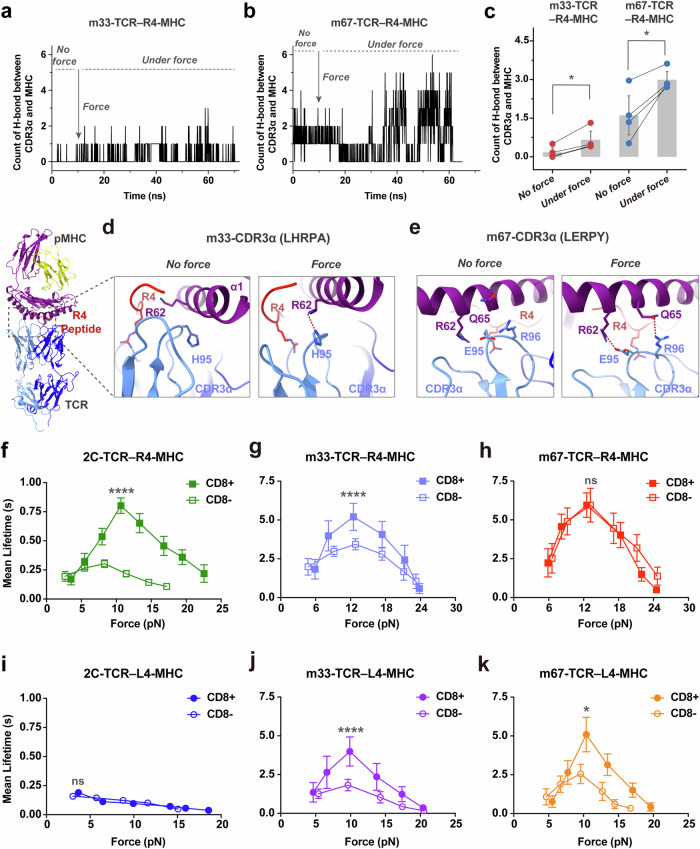
Strengthening force-dependent TCR–pMHC binding through TCR-CDR3α mutagenesis modulates CD8 enhancement power for TCR antigen recognition. **a**, **b** Time courses of the H-bond numbers between CDR3α motif of m33- (**a**) or m67-TCRs (**b**) and pMHC under cv-SMD simulations, with or without force. **c** Comparison of the counts of H-bonds, derived from analysis in **a**, **b**, in the presence or absence of force by paired *t*-tests. **d**, **e** Zoomed-in structural views of the contact between H-2K^b^ chain of R4-MHC and CDR3α of m33- (**d**) or m67-TCRs (**e**) in cv-SMD simulations, with mutations implemented through energy minimization and RoseTTAFold based on the 2C-TCR–H-2K^b^–R4 complex (PDB code: 1G6R). The mutated residues of CDR3α are respectively indicated on the top of each panel, and the H-bonds are indicated by red dashed lines. **f**–**k** Force-dependent mean lifetime curves of 2C-, m33- and m67-TCRs interactions with R4- (**f**–**h**) or L4-MHCs (**i**–**k**), when CD8 is absent or present. The peak bond lifetimes with or without CD8 were statistically analyzed by the Mann–Whitney *U*-test. The number of bond lifetime measurements per force curve for different TCR–pMHC or TCR–pMHC–CD8 pairs is summarized in Supplementary information, Table S2. All binned data points of force curves are presented as mean ± SEMs and summarized in Supplementary information, Tables S3, S4. Statistical significance was indicated as follows: ns not significant, *P < 0.05, ****P < 0.0001.

First, we performed molecular dynamics (MD) simulations to elucidate the dynamic structural mechanisms by which force regulates R4-MHC binding with 2C-, m33-, and m67-TCRs, respectively. Given that crystal structures of m33- and m67-TCRs complexed with R4-H-2K^b^ were not available, we employed RoseTTAFold to model their complex structures.^51^ The crystal structure of 2C-TCR with R4-H-2K^b^ (PDB code: 1G6R^52^) served as the initial template for these models. Comparing the dynamic structures of these three TCRs binding with R4- or L4-MHC under force,^27^ we observed that force could induce additional intermolecular binding sites for both m33- and m67-TCRs (Supplementary information, Fig. S3a–e). These binding sites not only include those found between TCRβ and MHC (e.g., N30 and E56 of TCRβ), similar to our previous findings for 2C-TCR,^27^ but also include sites on the m33- and m67-TCRs CDR3α motif and MHC-α_1_α_2_ domains (Fig. 2a−c). Force also induced the formation of an additional hydrogen bond (H-bond) (m33-TCRα-H95 and H-2K^b^-R62) for m33-TCR (Fig. 2d) and two additional ones (m67-TCRα-E95 and H-2K^b^-R62, m67-TCRα-R96 and H-2K^b^-Q65) for m67-TCR (Fig. 2e). Meanwhile, we examined the conformational changes in the m33- and m67-TCRs binding with R4-MHC in detail by analyzing the Root-Mean-Square Deviation (RMSD) of the TCR’s V domain. No significant structural changes were observed for R4-MHC binding with either m33- or m67-TCR (Supplementary information, Fig. S3f). Overall, mechanical force can enhance the bi-molecular interactions of m33- and m67-TCRs with R4-MHC by forming additional H-bonds between the TCR-CDR3α/TCR-CDRβ motifs and the H-2K^b^-α_1_α_2_ domains.

We then used a single-molecule ultra-stable biomembrane force probe (U-BFP)^53^ to characterize the force-dependent bond lifetimes of the three TCRs binding with R4- or L4-MHC over a wide range of forces in the absence of CD8 (Fig. 2f–k). Consistent with our prior findings,^27^ mechanical force prolonged the bond lifetimes of 2C-TCR–R4-MHC interactions (Fig. 2f), forming catch bonds with a peak bond lifetime of ~0.30 s at ~8.32 pN (Supplementary information, Table S3). In contrast, the interaction between 2C-TCR and L4-MHC exhibited a slip-bond behavior, where increasing force accelerated dissociation, resulting in a monotonic decrease in bond lifetimes (Fig. 2i; Supplementary information, Table S3). Interestingly, mechanical force induced catch bonds in m33-TCR interactions with both R4- and L4-MHCs. The m33-TCR–R4-MHC interaction exhibited a peak bond lifetime of ~3.43 s at ~12.85 pN, whereas the m33-TCR–L4-MHC interaction reached a peak bond lifetime of ~1.82 s at ~9.62 pN (Fig. 2g, j; Supplementary information, Table S3). Among the three TCRs, the m67-TCR exhibited the strongest 3D affinity in binding to R4-MHC. The m67-TCR–R4-MHC interaction demonstrated a force-prolonged peak bond lifetime of ~5.94 s, approximately 27-fold longer than that of the 2C-TCR–R4-MHC interaction (Fig. 2h; Supplementary information, Table S3). Force also strengthened the binding of m67-TCR to L4-MHC, with a peak bond lifetime of ~2.56 s, ~2.5-fold shorter than that of the m67-TCR–R4-MHC interaction (Fig. 2k; Supplementary information, Table S3). Notably, the forces corresponding to peak bond lifetimes consistently fell within a medium range of 9 to ~12 pN across the catch-bond curves. These findings demonstrate that mechanical force differentially regulates TCR–pMHC interactions, depending on the specific interaction between the peptide’s hotspot residue in the pMHC and the TCR-CDR3α domain.

**Fig. 3:**
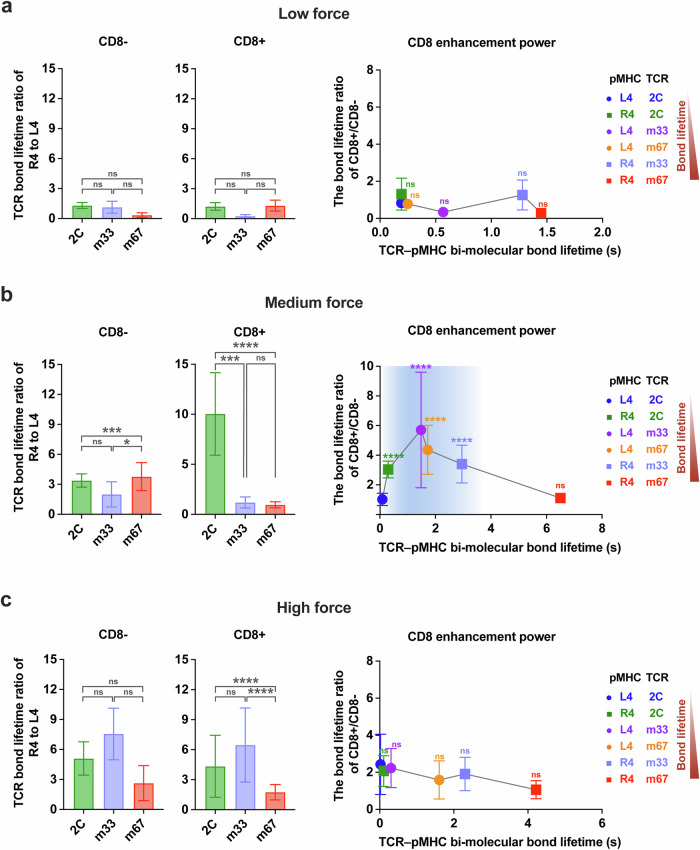
The specificity of TCR is regulated by CD8 cooperating with dynamic force. **a**–**c** Left, The analysis of TCR bond lifetime ratio (R4 to L4) in three force regimes: low (2–5 pN) (**a**), medium (9–12 pN) (**b**), and high (16–19 pN) (**c**) regimes, in the absence or presence of CD8. Right, the relationship between CD8 enhancement power and TCR–pMHC bi-molecular lifetimes in the low (**a**), medium (**b**), or high (**c**) force regimes. All data points are presented as mean ± SEM and summarized in Supplementary information Tables S6, S8. The statistical analyses for the left panels of **a**–**c** were conducted using unpaired student's *t*-tests, and the right panels of **a**–**c** were performed using the Mann–Whitney test, with 2C-TCR–L4-MHC as a control. Statistical significance was indicated as follows: ns not significant, *P < 0.05, ***P < 0.001, ****P < 0.0001.

We next examined the correlation between force-dependent bond lifetimes and TCR specificity. Since the variation of bond lifetimes with force magnitudes, we categorized the lifetimes into three distinct groups: low (2–5 pN), medium (9–12 pN) and high (16–19 pN) force regimes (Supplementary information, Table S5). We then calculated the relative change in lifetimes for the three TCRs interacting with R4- and L4-MHCs within these force regimes (Fig. 3a–c, left panels) without CD8. At the low force regime, the ratios of bond lifetimes for R4- vs L4-MHCs were approximately ~1.30-fold for 2C-TCR, ~1.13-fold for m33-TCR, and ~0.34-fold for m67-TCR (Fig. 3a, left panel; Supplementary information, Table S6). At the medium force regime, these differences increased to ~3.38-, ~1.99-, ~3.77-fold (Fig. 3b, left panel; Supplementary information, Table S6), indicating a more pronounced difference compared to the low force regime. At the high force regime, the differences further enhanced to ~5.10-, ~7.55-, ~2.63-fold (Fig. 3c, left panel; Supplementary information, Table S6), respectively. Despite these observations, none of these ratios were well correlated with the TCR specificities. Pearson correlation coefficients were *r* = 0.35 in the low force regime, *r* = –0.98 in the medium force regime, and *r* = –0.22 in the high force regime (Supplementary information, Fig. S4a, c and Table S7). These findings suggest that, in the absence of CD8, the relative changes in bond lifetimes across different force regimes are insufficient to predict TCR specificity.

### The ratio of TCR bond lifetimes in the medium force regime correlates well with TCR specificity in the presence of CD8

We examined the impact of CD8 on the correlation between TCR specificity and mechanical metrics, particularly on the ratio of TCR bond lifetimes under different force regimes. In the presence of CD8, mechanical force significantly strengthened the 2C-TCR–R4-MHC peak bond lifetime to ~0.80 s, nearly 3-fold longer than without CD8 (~0.30 s). This finding highlights the synergistic strengthening effect of CD8 and force (Fig. 2f; Supplementary information, Table S4). Similarly, the presence of CD8 further prolonged the peak bond lifetimes of m33-TCR interacting with R4- or L4-MHC from ~3.43 s or ~1.82 s without CD8 to ~5.20 s or ~3.99 s with CD8, respectively (Fig. 2g, j; Supplementary information, Table S4). In contrast, CD8 did not enhance the binding of 2C-TCR with non-stimulatory L4-MHC, as indicated by the near overlap of their force-dependent dissociation curves with and without CD8 (Fig. 2i; Supplementary information, Table S4). Notably, CD8 also failed to increase the lifetimes of the m67-TCR–R4-MHC binding across all force regimes, as the bond lifetime curves with and without CD8 almost overlapped, both reaching a maximum of ~5.95 s (Fig. 2h; Supplementary information, Table S4). However, for m67-TCR binding to L4-MHC, CD8 again exhibited an enhancing effect, increasing the peak lifetime from ~2.56 s without CD8 to ~5.09 s with CD8 (Fig. 2k; Supplementary information, Table S4), similar to its effect on 2C- and m33-TCRs with R4-MHC.

We then analyzed the relative changes in force-dependent bond lifetimes for the three TCRs binding to R4- or L4-MHC in the presence of CD8. The lifetime ratios for 2C-, m33- and m67-TCRs binding to R4- vs L4-MHCs varied significantly across force regimes (Fig. 3a–c, medium panels; Supplementary information, Tables S5, S6). Notably, the lifetime ratios in the medium force regime exhibited a strong positive correlation with TCR specificity (Supplementary information Fig. S4b, c and Table S7). These ratios were ~10.04-, ~1.19-, and ~0.96-fold for 2C-, m33-, and m67-TCRs, respectively, in the presence of CD8 (Fig. 3b, medium panel; Supplementary information Table S6). In contrast, ratios in low and high force regimes did not correlate with specificity (Fig. 3a, c, medium panels; Supplementary information, Fig. S4b, c and Table S7).

In conclusion, our findings demonstrate that in the presence of CD8, the ratio of bond lifetimes in the medium force regime correlates most closely with TCR specificity. Both m33- and m67-TCRs, despite exhibiting higher 3D affinities and stronger catch bonds with R4-MHC compared to 2C-TCR, were less effective at leveraging CD8 cooperation to enhance specificity under force. Their lifetime ratios in the medium force regime were nearly 10-fold lower than that of 2C-TCR when CD8 was present, underscoring the importance of moderate bond lifetimes in the medium force regime, in combination with CD8, for optimal TCR specificity.

### TCR specificity is dynamically regulated by the cooperation between CD8 and mechanical force

To understand how force-dependent TCR–pMHC bond lifetimes regulate CD8 engagement, we quantified the CD8 enhancement power across various TCR–pMHC pairs. CD8 enhancement power was defined as the ratio of bond lifetimes for TCR–pMHC interactions in the presence vs absence of CD8.

When comparing CD8 enhancement power across different force regimes, no differences were observed among TCR–pMHC pairs in the low force regime (Fig. 3a, right panel). This finding suggests that a force threshold above low levels is required for CD8 enhancement. Supporting this hypothesis, we observed a pronounced CD8 enhancement preference in the medium force regime, where TCR–pMHC bond lifetimes ranged from 0.30 to ~3.00 s (Fig. 3b, right panel). Within this range, CD8 enhancement increased bond lifetimes by ~3.04-fold for 2C-TCR–R4-MHC, ~5.70-fold for m33-TCR–L4-MHC, ~4.36-fold for m67-TCR–L4-MHC, and ~3.40-fold for m33-TCR–R4-MHC (Fig. 3b, right panel; Supplementary information, Table S8). Beyond this range, when the bi-molecular bond lifetime is excessively long (i.e., m67-TCR–R4-MHC) or short (i.e., 2C-TCR–L4-MHC), CD8 enhancement effect was not significant (Fig. 3a, c, right panels; Supplementary information, Table S8).

These findings collectively highlight that shaping TCR specificity requires not only optimal TCR–pMHC bond lifetimes in the medium force regime but also the cooperative engagement of CD8 under force. Given that T cells can exert molecular forces in the range of ~10–20 pN,^39,45^ we hypothesize that TCR–pMHC bond lifetimes in the medium force regime (~9–12 pN) represent the most relevant mechanical metric for determining CD8 enhancement power and TCR specificity. In summary, our findings reveal the kinetic mechanism by which TCR specificity is cooperatively regulated by three factors: the bond lifetime of TCR–pMHC interactions, the presence of CD8, and the magnitude of mechanical force. Future investigations will dissect the structural dynamics underlying this cooperative mechanism in greater detail.

### Dynamic structural mechanism of CD8’s selective cooperation for TCR specificity and off-target cross-reactivity

To elucidate the dynamic structural mechanism underlying how TCRs leverage CD8 and mechanical force to achieve high specificity, we constructed four structural models of TCR–pMHC–CD8 tri-molecular complexes: 2C-TCR–L4-MHC–CD8, 2C-TCR–R4-MHC–CD8, m33-TCR–R4-MHC–CD8 and m67-TCR–R4-MHC–CD8. We then performed constant-velocity steered molecular dynamics (cv-SMD) simulations to characterize the force-induced conformational changes in these complexes.

**Fig. 4:**
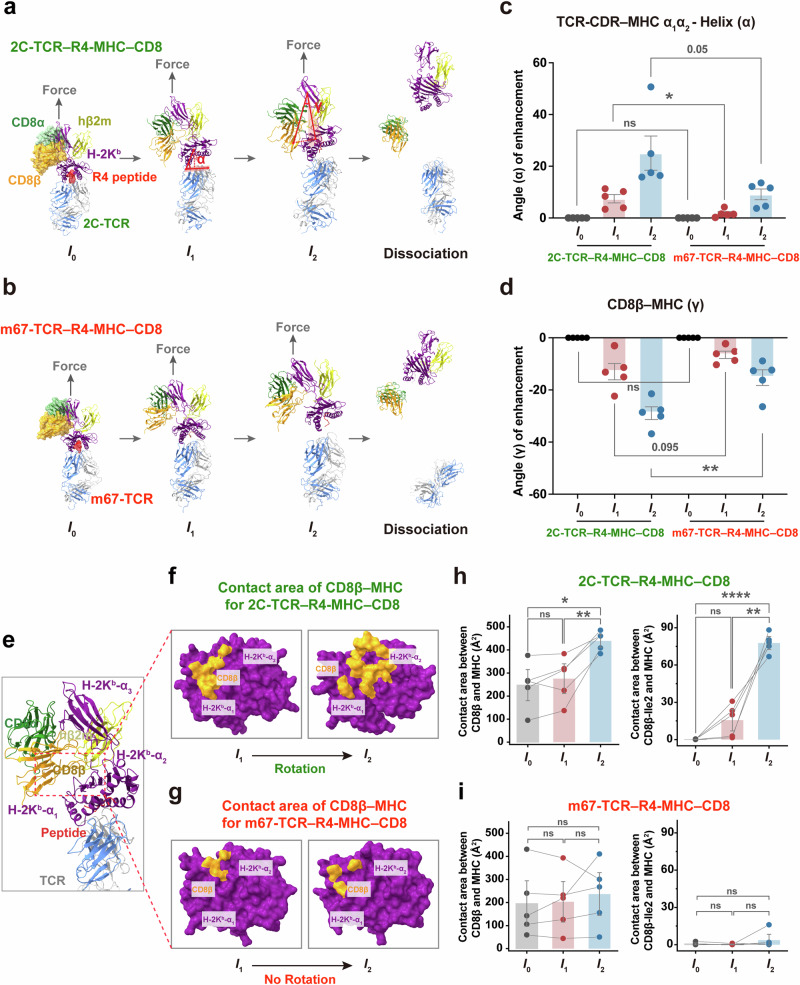
Mechano-regulated structural mechanism of CD8 enhancement for moderate or ultra-strong TCR–pMHC binding. **a**, **b** Sequential snapshots of cv-SMD simulations illustrating the dissociation of R4-MHC from 2C-TCR–CD8αβ (**a**) or m67-TCR–CD8αβ (**b**) modeled structural complexes under mechanical force pulling. **c**, **d** Force-induced angle changes of the TCR–pMHC (**c**) and the CD8β–pMHC interface (**d**) within 2C- and m67-TCRs systems in cv-SMD simulations. Detailed definitions of these angles are described in Supplementary information Fig. S6. **e**–**g** Structural footprint of CD8β (yellow) in complex with R4-MHC (purple) for 2C- (**f**) or m67-TCRs (**g**) systems, as depicted in two representative snapshots corresponding to states *I*_1_ and *I*_2_. Contact area between CD8β (or its Ile2 residue) and MHC under mechanical force in cv-SMD simulations for the 2C-TCR–R4-MHC–CD8 (**h**) and the m67-TCR–R4-MHC–CD8 (**i**) complexes. Error bars are ± SEMs. All statistical analyses were conducted using unpaired student's *t*-tests. Statistical significance was indicated as follows: ns not significant, *P < 0.05, **P < 0.01, ****P < 0.0001.

Our simulations revealed that mechanical force dynamically altered the conformation of the TCR–pMHC–CD8 complexes, transforming them from an initial state (*I*_0_) under zero force to two consecutive intermediate states (*I*_1_ under low force, and *I*_2_ under medium or high force) before the complete dissociation of the ternary complex (Fig. 4; Supplementary information, Fig. S5). These conformational transitions were initiated by the force-induced rotation of MHC-α_1_α_2_ domains relative to the TCR–pMHC binding interface, followed by the rotation of the β2m molecule relative to the MHC-α_3_ domain, and eventually the rotation of CD8αβ’s ligand binding domains (LBD) toward the MHC-α_1_α_2_ domains (Fig. 4; Supplementary information, Figs. S6, S7). The structural dynamics of these states varied based on the force-dependent bond lifetimes of the respective TCR–pMHC interactions.

For the 2C-TCR–R4-MHC–CD8 complex, which exhibited moderate catch-bond behavior (Fig. 2f), force initially enhanced the contact between the TCR and the pMHC, inducing a notable conformational change in the pMHC-α_1_α_2_ domains (Fig. 4a). This transition facilitated tighter TCR-CDRs binding to both the peptide and MHC-α_1_α_2_ domains, consistent with our previous finding for the 2C-TCR–R4-MHC interaction in the absence of CD8.^27^ In the *I*_1_ state, CD8 maintained contact with the MHC-α_3_ domain without significant rotation (Fig. 4a). As force increased, CD8’s LBD rotated to establish new contact with the MHC-α_1_α_2_ domains, defining the *I*_2_ state, followed by dissociation of the complex (Fig. 4a). Key structural changes between the *I*_1_ and *I*_2_ states included increases in the TCR–pMHC rotation angle α by ~7.45° (*I*_1_) and ~25.07° (*I*_2_) (Fig. 4c; Supplementary information, Fig. S7a), decreases in β2m–MHC-α3 rotation angle β by ~3.50° (*I*_1_) and ~9.14° (*I*_2_) (Supplementary information, Fig. S7b), and decreases in the CD8–MHC angle γ by ~12.99° (*I*_1_) and ~28.92° (*I*_2_) (Fig. 4d; Supplementary information, Fig. S7c). These changes corresponded to an ~1.8-fold increase in the contact areas between CD8β and MHC, from ~248 Å^2^ (*I*_0_) to ~439 Å^2^ (*I*_2_) (Fig. 4e–g). Additionally, the hydrophobic residue CD8β-Ile2 exhibited force-induced interaction with the MHC-α_1_α_2_ domains. The contact area between CD8β-Ile2 and MHC increased from ~0 Å² in the *I*_0_ state to ~78 Å² in the *I*_2_ state (Fig. 4h). This finding highlights CD8β-Ile2 as a critical residue mediating force-enhanced interactions at the CD8–MHC interface.

For the m33-TCR–R4-MHC–CD8 complex, similar force-driven rotations of pMHC and CD8-LBD were observed, albeit to a lesser extent than in the 2C-TCR system (Supplementary information, Fig. S5a). Angle α increased by ~11.52° (*I*_1_), and ~23.08° (*I*_2_) (Supplementary information, Fig. S7a), angle β decreased by ~3.15° (*I*_1_), and ~4.86° (*I*_2_) (Supplementary information, Fig. S7b), and angle γ decreased by ~7.00° (*I*_1_), and ~16.03° (*I*_2_) (Supplementary information, Fig. S7c). The CD8β–MHC contact area increased modestly from ~186 Å² to ~289 Å², with a smaller contribution from CD8β-Ile2 (contact area ~30–60 Å² in *I*_2_ states) compared to the 2C-TCR complex (Supplementary information, Fig. S5b). This weaker interaction explains the reduced CD8 enhancement power observed for m33-TCR binding to R4-MHC.

Conversely, the 2C-TCR–L4-MHC–CD8 complex exhibited negligible force-induced conformational changes. Neither substantial rotation of CD8’s LBD toward MHC-α_1_α_2_ domains nor increased contact area between CD8β and MHC was detected. Particularly, the contact area between CD8β-Ile2 and MHC in the *I*_2_ state was nearly 0 Å^2^. Consequently, this complex rapidly dissociated without forming a stable *I*_2_ state (Supplementary information, Fig. S5c, d).

For the m67-TCR–R4-MHC–CD8 complex, while force enhanced the interactions of TCR-CDRs with the pMHC, it failed to induce significant rotation of the MHC-α_1_α_2_ domains or CD8-LBD (Fig. 4b). The angles α, β and γ did show minimal changes in force-induced states. Angle α increased by ~1.74° (*I*_1_) and ~9.14° (*I*_2_) (Fig. 4c); angle β reduced by ~1.93° (*I*_1_), and ~4.08° (*I*_2_) (Supplementary information, Fig. S7b); and angle γ reduced by ~6.53° (*I*_1_) and ~15.31° (*I*_2_) (Fig. 4d). Additionally, force could not induce a significant increase in the contact area between CD8β and MHC, from ~196 Å^2^ (*I*_0_) to ~203 Å^2^ (*I*_1_) to ~237 Å^2^ (*I*_2_), or between CD8β-Ile2 residue and MHC, from ~1 Å^2^ (*I*_0_) to ~0 Å^2^ (*I*_1_) to ~4 Å^2^ (*I*_2_) (Fig. 4e, g, i). Together, these results underscore the inability of strong TCR–pMHC binding to facilitate effective CD8-mediated stabilization.

Collectively, our findings reveal a dynamic structural mechanism by which force-induced rotational conformational changes of pMHC and CD8-LBD regulate TCR–pMHC interactions. The cooperative action of force and CD8 preferentially enhances TCR–pMHC pairs with moderate catch-bond strength, optimizing specificity. However, excessively strong or weak TCR–pMHC binding under force impairs the force-induced rotations of CD8 and MHC-α_1_α_2_ domains towards the TCR, resulting in reduced CD8 enhancement by not forming an essential intermediate state *I*_2_. This dynamic structural mechanism provides a straightforward explanation for the off-target cross-reactivity of engineered high-affinity TCRs.

### Blocking force-enhanced CD8–MHC binding impairs TCR specificity

**Fig. 5:**
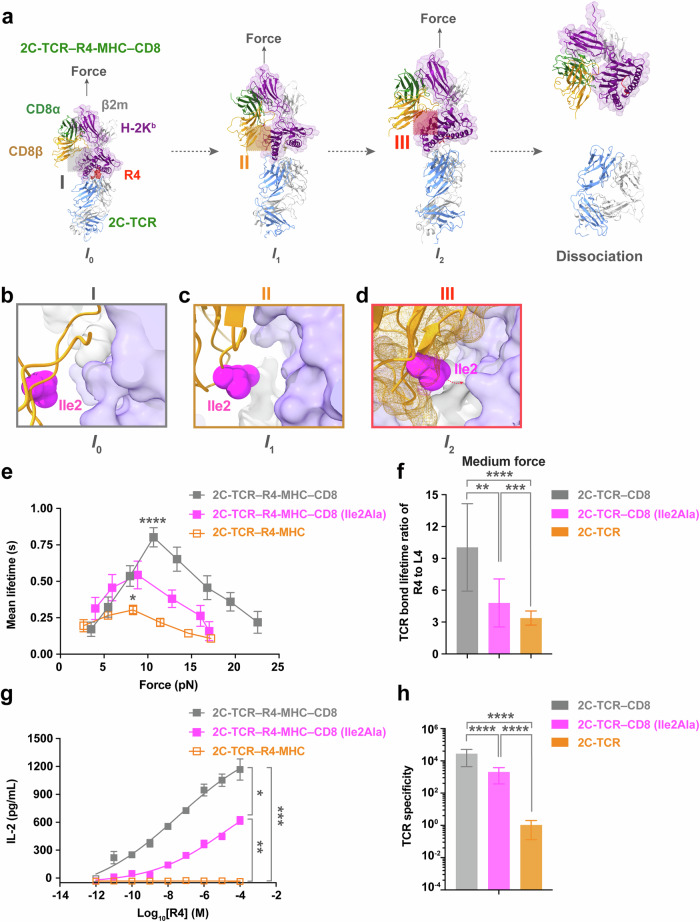
Blocking force-enhanced interactions at the CD8–MHC binding interface impairs TCR specificity. **a** Sequential conformational change of CD8–pMHC interface for 2C-TCR–R4-MHC–CD8αβ complex during cv-SMD simulations. **b**–**d** Zoomed-in snapshots of dynamic force exerted onto 2C-TCR–R4-MHC (light purple and gray surface)–CD8 (orange color, meshed surface and ribbon) complex structures at three key states: an initial state *I*_0_ **b**, an unstable intermediate state *I*_1_ **c**, and a stable intermediate states *I*_2_ **d**. These states are characterized by the hydrophobic contact areas between CD8β-Ile2 (pink ball) and MHC. **e** Force-dependent mean lifetime curves for the interactions of 2C-TCR with R4-MHC, in the presence of WT CD8, or CD8 (Ile2Ala), or in the absence of WT CD8, respectively. The lifetime curves for the presence and absence of WT CD8 from Fig. 2f are replotted in Fig. 5e for comparison. The peak bond lifetimes with WT CD8, or CD8 (Ile2Ala) vs without CD8 were statistically analyzed. Single-molecule U-BFP assay ensured consistent conditions across all measurements, with each force value derived from >100 single-molecule bond lifetime measurements. The number of bond lifetime measurements per force curve for different TCR–pMHC or TCR–pMHC–CD8 pairs is summarized in Supplementary information, Table S2. All binned data points of force curves are presented as mean ± SEMs and summarized in Supplementary information, Tables S3, S4. **f** The analysis of TCR lifetime ratio (R4 to L4) in the presence of WT CD8, CD8 (Ile2Ala), or in the absence of WT CD8, respectively. The lifetime ratio (R4 to L4) for the presence and absence of WT CD8 from Fig. 3b was replotted in Fig. 5f for comparison. **g**, **h** IL-2 production of 2C hybridomas cells and the analysis of TCR specificity in the presence of WT CD8, CD8 (Ile2Ala), or in the absence of WT CD8, respectively. All data points are presented as mean ± SEMs and summarized in Supplementary information, Tables S6, S8. The statistical analyses for the panels **e**, **g** were conducted using the Mann–Whitney test, and the panels **f**, **h** were performed using unpaired *t*-tests. Statistical significance was indicated as follows: *P < 0.05, **P < 0.01, ***P < 0.005, ****P < 0.0001.

Based on our simulation findings (Fig. 5a–d), we hypothesized that disrupting force-induced CD8–MHC contact might impair TCR specificity. To validate this, we mutated CD8β-Ile2 to alanine (CD8β-Ile2Ala) and examined its impact on CD8’s role in regulating TCR specificity. We expressed this mutated CD8 on 2C hybridoma cells and ensured that its expression level matched that of the wild-type (WT) CD8 to avoid the interference from different expression levels (Supplementary information, Fig. S8a, b). As expected, this single mutation reduced the force-dependent bond lifetimes of the 2C-TCR–R4-MHC–CD8 interaction by ~1.5-fold (i.e., its peak lifetime was reduced from ~0.80 s to ~0.54 s) (Fig. 5e; Supplementary information, Table S4), and halved the lifetime ratio of R4- to L4-MHC binding (i.e., from ~10.04 for WT CD8 to ~4.81 for mutated CD8) (Fig. 5f; Supplementary information, Table S6). It also reduced CD8 enhancement power by ~2 times (Supplementary information, Table S8), resulting in a ~9-fold decrease in TCR specificity (i.e., ~6.55 × 10^4^ for WT CD8, and ~7.07 × 10^3^ for CD8β-Ile2Ala) (Fig. 5g, h; Supplementary information, Fig. S8c and Table S6).

### High-affinity TCR engineering impairs antigen specificity in human TCRs

**Fig. 6:**
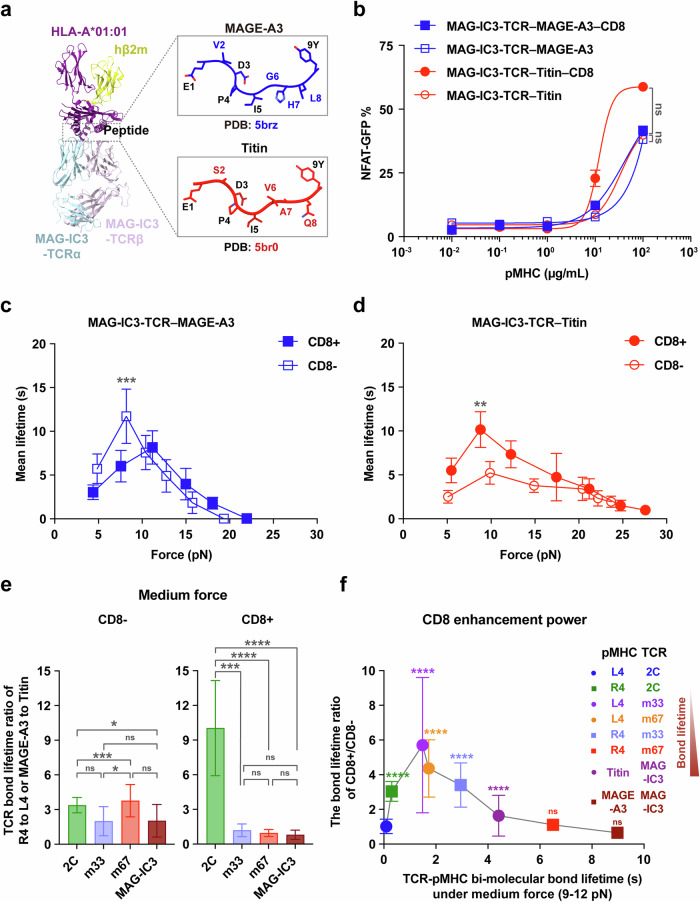
Human high-affinity TCR engineering impairs antigen specificity. **a** Structural overview of MAG-IC3-TCR (light blue of TCRα and misty rose of TCRβ) bound to the MAGE-A3-HLA-A*01:01 or Titin-HLA-A*01:01 complex. The components of MAGE-A3-derived peptide (EVDPIGHLY), Titin-derived peptide (ESDPIVAQY), HLA-A*01:01 and β2m are depicted in blue, red, purple and yellow, respectively. **b** NFAT-GFP population of MAG-IC3 T cells expressing CD8 or not, stimulated by a series of concentrations of MAGE-A3-HLA-A*01:01 or Titin-HLA-A*01:01 protein. The presented data represent one of three independent experiments. The statistical analyses were performed using the Mann–Whitney test. **c**, **d** Force-dependent mean lifetime curves of MAG-IC3-TCR interactions with MAGE-A3 (**c**) or Titin (**d**) when CD8 is absent or present. The number of bond lifetime measurements per force curve for different TCR–pMHC or TCR–pMHC–CD8 pairs is summarized in Supplementary information, Table S2. All binned data points of force curves are presented as mean ± SEM and summarized in Supplementary information, Tables S3, S4. The statistical analyses were performed using the Mann–Whitney *U*-test. **e**, **f** The analysis of TCR bond lifetime ratio and CD8 enhancement power in the medium force regime reveals low specificity and weak enhancement for engineered high-affinity TCRs. Bond lifetime ratios and CD8 enhancement power for 2C-, m33-, and m67-TCRs from Fig. 3b were replotted for comparison. All data points are presented as mean ± SEM and summarized in Supplementary information, Tables S6, S8. The statistical analyses for the panel **e** were conducted using unpaired studen's *t*-tests, and those for the panel **f** were performed using the Mann–Whitney *U*-test, with 2C-TCR–L4-MHC as a control. Statistical significance was indicated as follows: ns not significant, *P < 0.05, **P < 0.01, ***P < 0.001, ****P < 0.0001.

To further validate the aforementioned findings, we examined how high-affinity engineering of human TCRs impairs their antigen specificities. As a model system, we used the human MAG-IC3-TCR, which was originally engineered through affinity maturation to enhance its binding to the tumor-associated antigen MAGE-A3-derived epitope (EVDPIGHLY) presented by human leukocyte antigen A-01:01 (HLA-A*01:01).^15,26^ However, the MAG-IC3-TCR was later found to cross-react with a self-antigen derived peptide (e.g., ESDPIVAQY, derived from Titin), presented on HLA-A*01:01, which led to severe off-target toxicity in human (Fig. 6a, b).^17,18,26^ Despite this, how the specificity of high-affinity TCRs is regulated by mechanical force and CD8 remains unclear.

We observed that the MAG-IC3 TCR displayed exceptionally long bond lifetimes under force in the absence of CD8, with the peak lifetime at ~11.72 s for MAGE-A3 under ~8.2 pN and ~5.22 s for Titin under ~9.88 pN (Fig. 6c, d; Supplementary information, Table S3). Interestingly, CD8 reduced the peak bond lifetime for the MAGE-A3 interaction from ~11.72 s to ~8.16 s, indicating minimal CD8 enhancement (Fig. 6c; Supplementary information, Tables S3, S4). CD8 nearly doubled the bond lifetime for the Titin interaction, from ~5.22 s to ~10.15 s (Fig. 6d; Supplementary information, Tables S3, S4), suggesting significant CD8 enhancement for off-target interactions.

Integrating data from both the 2C-TCR and MAG-IC3-TCR systems, we further confirmed that the TCR–pMHC bond lifetimes in the medium force regime critically regulate TCR specificity (Fig. 6e; Supplementary information, Tables S5, S6). When TCR–pMHC binding was either too strong or too weak, CD8’s contribution was minimal or absent (Fig. 6f; Supplementary information, Table S8). High-affinity engineered TCRs with prolonged bond lifetimes exhibited reduced CD8-mediated specificity enhancement, predisposing them to off-target interactions. Conversely, TCRs with optimal binding strengths to cognate antigens under force efficiently engaged CD8, improving specificity and mitigating off-target cross-reactivity.

### Predicted TCR recognition signals from force-dependent TCR–pMHC binding kinetics

Building on the insights from this study, we explored whether TCR–pMHC recognition signals could be accurately predicted using force-dependent kinetics. To achieve this, we integrated in situ TCR–pMHC binding characteristics, including 2D effective binding affinities $({A}_{{{{\rm{c}}}}}{K}_{{{{\rm{a}}}}})$,^21,22^ bond lifetimes under force, and CD8 enhancement power from this study. These parameters were used to construct comprehensive TCR–pMHC kinetics-function maps to predict TCR recognition potential.

**Fig. 7:**
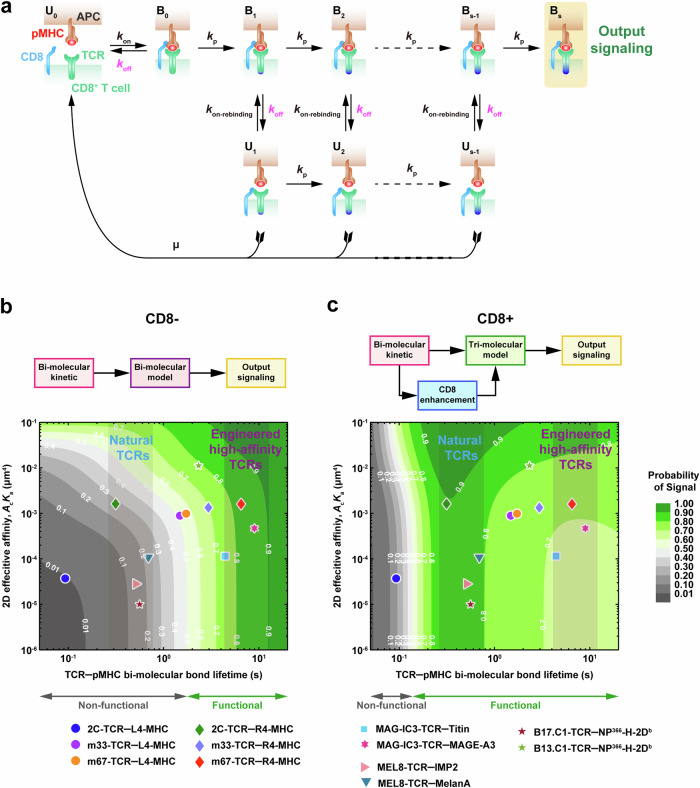
The 2D affinities and force-dependent bond lifetimes of TCR–pMHC bi-molecular interactions shape TCR antigen recognition. **a** Schematic diagram of the force-dependent kinetic proofreading model of TCR triggering in the presence of CD8. **b**, **c** Mapping of TCR–pMHC 2D affinities and bond lifetimes in the medium force regime onto kinetics-function maps, in the absence (**b**) or presence of CD8 (**c**) Data presented include both our experimental results and previously published data. The bond lifetime data for NP^366^-H-2D^b^-specific canonical B13.C1-TCR and B17.C1-TCR were from a previous study.^56^ All other data were from our own experiments.

To develop these maps, we constructed two kinetic proofreading (KPR) models for TCR–pMHC interactions,^54^ the bi-molecular model (TCR–pMHC interactions without CD8) (Supplementary information, Fig. S9a) and the tri-molecular model (TCR–pMHC interactions with CD8) (Fig. 7a) (see Materials and Methods for detailed modeling description). Simulations were implemented by systematically changing TCR–pMHC bond lifetimes (${t}_{{{{\rm{b}}}}}=1/{k}_{{{{\rm{off}}}}}$ from 10^–2^ to 10 s) and 2D effective binding affinity (${A}_{{{{\rm{c}}}}}{K}_{{{{\rm{a}}}}}$, from 10^–6^ to 10^–1^ μm^4^), while keeping other parameters constant or being estimated from lifetime-dependent curves (see detailed description in Materials and Methods). The resulting simulated signals were quantified as the probability of a TCR–pMHC complex reaching the final recognition state within 10 s.

Validation of these models using functional measurements of six 2C-TCR pairs showed strong correlations between predicted productive signals in the medium force regime and IL-2 secretion (*R* = 0.89, *P* = 0.033 for bi-molecular model; *R* = 0.94, *P* = 0.017 for tri-molecular model, Supplementary information, Fig. S9b, c). The kinetics-function maps revealed a non-functional region (signal probability < 0.5, gray regions in Fig. 7b, c), which encompassed TCR–pMHC pairs (e.g., the 2C-TCR–L4-MHC pair) with short bond lifetimes, regardless of CD8 presence. In contrast, for TCR–pMHC with moderate bond lifetimes (e.g., 2C-TCR–R4-MHC, green diamond in Fig. 7b, c), CD8 enhancement shifted these interactions from non-functional (gray regions, Fig. 7b) to functional (green regions, Fig. 7c). Most natural TCRs clustered within this optimal region, demonstrating their reliance on CD8 for improved recognition. Conversely, TCR–pMHC pairs with excessively long bond lifetimes hindered CD8 enhancement, suppressing recognition signals (e.g., the m67-TCR–R4-MHC pair, red diamond moving from dark green region in Fig. 7b to vivid green region in Fig. 7c).

To further validate these maps, we conducted additional U-BFP experiments to characterize the force-dependent binding kinetics of the natural MEL8-TCR interacting with two tumor-associated antigen peptides, MelanA and IMP2 (Supplementary information, Fig. S10a–d).^55^ These TCR pairs as well as other published TCR–pMHC pairs, including human tumor-specific TCRs (e.g., MAG-IC3-TCR with its parent TCR’s cognate peptide from MAGE-A3 and the cross-reactive peptide from Titin^15,26^) and mouse viral-specific TCRs (NP^366^-H-2D^b^-specific canonical B13.C1-TCR and B17.C1-TCR),^56^ were then incorporated in the kinetic-function maps (Fig. 7b, c). In the absence of CD8, predicted signals for all tested TCR–pMHC pairs closely matched functional observations.^55,56^ Specifically, NP^366^-B17.C1-TCR (plum star) and MEL8-TCR exhibited non-functional signals when recognizing MelanA (dark blue bottom triangle) and IMP2 (pink right triangle), respectively. In contrast, NP^366^-B13.C1-TCR (green star), MAG-IC3-TCR recognizing MAGE-A3 (rose-red star), and Titin peptides (light-blue square) demonstrated functional signals (Fig. 7b). Analysis of TCR signals in the presence of CD8 revealed distinct patterns. For natural TCRs such as NP^366^-B17.C1-TCR and MEL8-TCR recognizing MelanA and IMP2, CD8 enhancement converted non-functional interactions into functional ones, shifting these interactions to green regions in the map (Fig. 7c). However, high-affinity NP^366^-B13.C1-TCRs and engineered TCRs like MAG-IC3-TCR (binding MAGE-A3 and Titin peptides) already exhibited functional signals without CD8, and CD8 enhancement did not further amplify their recognition signals (Fig. 7c). These results mirrored the behavior of m33-TCR–R4-MHC and m67-TCR–R4-MHC pairs. Importantly, TCRs with low specificity, such as MAG-IC3-TCR, consistently fell into regions where CD8 provided minimal or inhibitory enhancement, underscoring the utility of these maps in identifying off-target risks (Fig. 7b, c).

These findings highlight the dynamic interplay among TCR–pMHC bi-molecular bond lifetimes, CD8 involvement, and mechanical force in shaping TCR specificity. The kinetics-function maps provide a robust framework for predicting TCR functionality and optimizing TCR engineering. By incorporating force-dependent kinetics and CD8 effects, these maps offer valuable guidance for mitigating off-target risks and designing safer, more effective engineered TCRs.

## Discussion

Our study reveals that the optimal specificity of TCRs emerges from the dynamic interplay among TCR–pMHC bond lifetimes, the coreceptor CD8, and mechanical force. By examining the structural and kinetics underpinnings of natural TCRs’ antigen recognition, we elucidated why engineered high-affinity TCRs frequently exhibit off-target cross-reactivity. To advance understanding in this area, we developed kinetics-function maps that integrate in situ 2D binding affinity, force-dependent TCR–pMHC bond lifetimes, and CD8-mediated enhancement. These maps also incorporate kinetic data from published human and murine TCR–pMHC interactions, providing a potentially robust and valuable framework for evaluating TCR specificity and guiding the design of therapeutic TCRs with high precision and minimal off-target effects.

Previous studies have reported inconsistencies in correlating TCR specificity with both 3D and 2D binding affinities of TCR–pMHC interactions.^23,27^ These studies also highlight that mechanical force can induce catch-bond formation between TCRs and pMHCs, enhancing TCR specificity, triggering T cell activation,^23,27,57^ and forming a mechanical feedback loop with CD8 to drive TCR signaling and negative selection in the thymus.^39^ However, the precise role of mechanical force in shaping TCR specificity, particularly how natural or engineered high-affinity TCRs utilize CD8 under force, remains poorly understood. Additionally, the optimal binding characteristics for evaluating TCR specificity have not been clearly defined. Our findings demonstrate that force-dependent kinetics provide critical insights into TCR specificity. Key factors include in situ association rates influenced by the mechanical constraints of the 2D plasma membrane of a cell, force-dependent bi-molecular TCR–pMHC bond lifetime (dissociation rate), and CD8 enhancement. Notably, we identified an optimal range of TCR–pMHC bond lifetimes in the medium force regime that facilitates CD8 cooperation, and enhances TCR specificity (Fig. 3b). This optimal lifetime region parallels the “peak theory” from recent CAR-T cell research, which emphasizes an optimal pre-ligand engagement signal window for CAR-T functionality.^58,59^ While the CAR-T model focuses on tonic signaling before ligand binding, our findings emphasize the post ligand engagement process. These models together may offer a unified perspective on regulating T cell specificity, sensitivity, and memory.

We developed force-dependent KPR models that integrate elements of serial triggering, dynamic catch bonds, and force-sensitive antigen recognition into the kinetics-function maps (Fig. 7b, c). This framework explains TCR specificity as a prominent property arising from the interplay of biophysical and biochemical factors. By incorporating key factors such as in situ bi-molecular TCR–pMHC binding affinity, force-dependent dissociation rates of TCR–pMHC interactions, and CD8 enhancement, the maps identify the optimal range of TCR specificity and differentiate between natural and engineered high-affinity TCRs. Our findings indicate that TCR specificity cannot be attributed to any single kinetic parameter but instead emerges from the integration of these factors. This integration aligns well with previous kinetic models. One is the serial triggering model, which suggests that a limited number of pMHC complexes can sequentially activate multiple (~200) TCRs.^32^ It emphasizes that an optimal, short-lived TCR–pMHC bond is more effective for T cell activation, whereas excessively long-lived bonds may reduce efficacy. This observation is consistent with our finding that engineered high-affinity TCRs with ultra-long-lived bond lifetimes impair T cell activation. The other is the dynamic catch-bond model, which highlights the importance of rapid catch-bond formation during the first minute of ligand engagement.^57^ These bonds are critical for effective TCR activation and require both fast ligand association rates and force-dependent dissociation rates. By integrating these principles, our model underscores the importance of combinatorial force-dependent kinetics in defining the early stages of TCR activation and specificity.

Our study identifies three modes of CD8 cooperation under mechanical force, each regulating TCR specificity differently. Firstly, when TCR–pMHC binding strength is weak, insufficient mechanical engagement between TCR and pMHC, as seen in the 2C-TCR–L4-MHC complex, prevents CD8 engagement due to rapid pMHC dissociation (Supplementary information, Fig. S11). Secondly, when force-dependent TCR–pMHC bond lifetimes are moderate, in systems like 2C-TCR with R4-MHC, mechanical forces strengthen TCR-CDRs interactions with peptide hotspots, which then induce conformational changes that promote CD8 engagement, enabling effective TCR signaling (Supplementary information, Fig. S11). We speculate that this sequential mechanical adjustment may extend the CD8 ectodomain, particularly its stalk region, making it more upright on the T cell membrane. Consequently, CD8’s C-terminal region, along with its associated Lck kinase, may move closer to the TCR–CD3 complex, effectively triggering TCR signaling.^39,60^ Thirdly, when TCR–pMHC bonds are ultra-long-lived under force, TCRs with excessively strong force-dependent bonds, such as m67-TCR with R4-MHC, exhibit restricted force-induced MHC rotation and CD8 engagement (Supplementary information, Fig. S11). This mechanical restriction may further impair the ability of CD8-coupled Lck kinases to interact with a proximal TCR–CD3 complex, limiting Lck activation and diminishing TCR sensitivity and downstream signal triggering. In addition, we speculate that CD4 may operate through a similar mechanism to regulate TCR specificity. Investigating this possibility in future may provide deeper molecular insights into coreceptor-mediated TCR antigen recognition.^61–63^

Our framework provides valuable insights into the structural basis of off-target cross-reactivity observed with engineered high-affinity TCRs, such as m67-TCR or MAG-IC3-TCR. These TCRs exhibit ultra-long-lived bonds that disrupt the dynamic conformational changes required for effective TCR–pMHC–CD8 interactions. This rigid binding architecture may enable non-specific recognition of self-antigens, compromising therapeutic safety and efficacy.^14–18,26,64^ To mitigate these clinical risks, strategies like targeted genetic modifications within TCR-CDRs have been proposed to fine-tune catch-bond formation and reduce off-target effects.^26^ While this strategy holds promise, it remains unclear whether the formation of TCR–pMHC catch or slip bonds alone is sufficient to define and ensure optimal TCR specificity. Our study suggests that merely inducing catch bonds may be insufficient and that optimal TCR specificity requires preserving the dynamic conformational flexibility of TCR–pMHC interactions.

Understanding the dynamic mechano-chemistry of TCR specificity is pivotal for optimizing therapeutic TCRs. By elucidating the interplay between TCR–pMHC bond dynamics, CD8 engagement, and mechanical force, our findings provide a quantitative framework for designing safer, more effective TCR-based therapies. To our knowledge, these insights lay the groundwork for advancing next-generation TCR-engineered therapies with improved precision and safety profiles.

While our study provides key insights into the mechano-chemical basis of TCR–pMHC interactions, it is important to acknowledge its limitations. First, we only analyzed a small subset of TCRs relative to the vast diversity of natural TCR repertoires.^8^ Variability in TCR behavior, as reported in earlier studies,^65^ underscores the need for broader investigations across different TCRs and antigens. Second, factors beyond TCR–pMHC binding strength, including tissue origin, membrane composition, and subcellular microenvironment, may influence catch-bond dynamics. For instance, evidence indicates differences in catch-bond dynamics between naïve T cells and established T cell lines. Variations in membrane cholesterol distribution have also been shown to impact receptor–ligand binding strength.^66–68^ Structural components of the TCR–CD3 complex may further modulate TCR specificity and function. Features such as the F-G loop of the TCRβ chain or the transmembrane domains of TCRαβ may also influence TCR conformation and antigen recognition.^48–50,69–71^ Recent work by Tom Walz’s group demonstrated a closed and bent conformation of TCRαβ ectodomains when associated with CD3 components in lipid nanodiscs, contrasting with the open and upright structure observed in detergent-based systems.^72^ This distinct conformation suggests that lipid membrane may impact TCR antigen recognition. Finally, our MD simulations did not consider the TCR transmembrane domains or the influence of other CD3 chains. This limitation may restrict our understanding of how the complete TCR–CD3 complex operates within the lipid membrane. Future studies should include these components to refine the model of TCR antigen recognition further.

## Materials and methods

### Construction of plasmids and T cell lines

The cDNA of the *2C-TCR*, *MAG-IC3-TCR*, and *MEL8-TCR* were synthesized commercially (Sangon Biotech, China), integrated using the IRES system, and cloned into the pHAGE lentiviral vector using the Hieff Clone Plus One-Step Cloning Kit (YEASEN, China). To generate mutant TCRs, the 2C-TCR CDR3α (VSGFASAL) was altered to create m33-TCR CDR3α (VSLHRPAL) and m67-TCR CDR3α (VSLERPYL). Mouse *CD8α* and *CD8β* cDNA were purchased from Sino Biological, Inc. (China) and cloned into the pHAGE vector using the IRES system. Human *CD8α* and *CD8β* cDNA were obtained from a peripheral blood mononuclear cell cDNA library. The preparation of T hybridoma cells has been described in the previous papers.^27^ In brief, the *α* and *β* genes of the 2C-, m33-, and m67-TCRs were transduced into 58α-β- hybridomas deficient in mouse-TCR expression, while the genes of *MAG-IC3-TCR* and *MEL8-TCR* were transduced into J76-NFAT-GFP Jurkat cells deficient in human-TCR expression, all using a lentiviral packaging system. Transduced cells were stained with anti-mouse TCRβ antibody (clone: H57-597, BD) or anti-human TCRαβ antibody (clone: IP26, BioLegend) and sorted for TCR-positive cells via fluorescence-activated cell sorting (FACS) using a Beckman MoFlo Astrios EQ system. Furthermore, mouse *CD8α* and *CD8β* genes were transduced into 58α-β- hybridomas and 2C-, m33-, and m67-TCR hybridomas, while human *CD8α* and *CD8β* genes were transduced into J76-NFAT-GFP cells expressing MAG-IC3-TCR and MEL8-TCR. Following transduction, cells were stained with anti-mouse CD8β antibody (clone: eBioH35-17.2, eBioscience) or anti-human CD8β antibody (clone: SIDI8BEE, eBioscience) and sorted for CD8β-positive cells using FACS. All cell lines were cultured at 37 °C in a humidified atmosphere with 5% CO_2_. The culture medium used was RPMI 1640 supplemented with 10% fetal bovine serum (YEASEN, China), 100 U/mL penicillin, and 100 µg/mL streptomycin (YEASEN, China).

### TCR inclusion body expression

DNA sequences encoded the ectodomains of 2C-, m33-, or m67-TCR-α and -β chains were optimized for the *Escherichia coli* (*E. coli*) expression system and cloned into pET-22b (+) vector (Beijing Tsingke Biotech Co., Ltd), between *Nde*1 and *Xho*1 restriction enzyme cleavage sites. A hexahistidine tag (His-tag) was introduced at the C-terminus of TCR-α chain for subsequent nickel column purification. Inclusion body production was described before.^73^ Briefly, recombinant TCRα and TCRβ were expressed in *E. coli* BL21 (DE3). A final concentration of 1 mM IPTG was added to induce expression of inclusion bodies for ~4 h at 37 °C when the growing cell density reached an OD600 value equal to 0.6–0.8. Cell pellets were suspended and lysed in the extraction buffer (50 mM Tris-HCl, 100 mM NaCl, 1% Triton X-100, pH 8.2) with fresh lysozyme, Dnase-I, and PMSF. After cell lysis via sonification, inclusion bodies were collected at 8000 rpm for 10 min and washed with wash buffer (50 mM Tris-HCl, 20 mM EDTA, pH 8.0).

### TCR refolding and purification

To refold the soluble TCR proteins, the total amount of 120 mg inclusion bodies of TCRα and TCRβ (mass ratio, 1.5:1) were diluted in the refolding buffer (100 mM Tris-HCl, pH 8.0, 0.4 M arginine, 0.5 mM oxidized glutathione, 1.5 mM reduced glutathione, 2 mM EDTA, 4 M urea, and 0.2 mM PMSF) in a volume of 400 mL over 24 h at 4 °C, as reported previously.^74,75^ The refolding solution was then dialyzed multiple times against 10 mM Tris-HCl (pH 8.0) at 4 °C by a 6–8 kDa molecular mass cut-off dialysis membrane (Cat. 132670, Spectrum). After dialysis, proteins with His-tag were affinity-enriched by a nickel column. The eluted proteins from nickel column were concentrated to a small volume and then loaded onto the Hiload 16/600 Superdex 75 pg prepacked column (Cytiva) for molecules separation in the running buffer composed of 20 mM Tris-HCl, 100 mM NaCl at pH 8.0. The Superdex 200 Increase 10/300 GL prepacked column (Cytiva) was applied for the final purification and buffer exchange. All purifications were performed on ӒKTA goTM chromatography system and analyzed via UNICORN 7.

### Purification of biotinylated pMHC proteins

The procedures of pMHC-I protein purification have been described in detail in the previous paper.^27^ In brief, the extracellular sequence of the heavy chain of mouse H-2K^b^ (amino acids 1–274), or human HLA-A*01:01 (amino acids 1–274), or human HLA-A*02:01 (amino acids 1–274) with a C-terminal AVI-tag (GLNDIFEAQKIEWHE) and the light chain of human β2m (amino acids 1–99) were successfully constructed into the pET28a vector. The inclusion bodies were induced and purified by the *E. coli* expression system. The purified inclusion bodies of mouse H-2K^b^, human HLA-A*01:01, human HLA-A*02:01, and human β2m, were denatured in 8 M urea and subsequently renatured in folding buffer (400 mM l-arginine and 100 mM Tris, pH 8.0, 2 mM EDTA, 5 mM reduced glutathione, 0.5 mM oxidized glutathione) and reassembled with the corresponding antigen peptide (R4, L4, MAGE-A3, or Titin) (QYAOBIO, China) to form a pMHC complex. The folded pMHC protein was obtained by concentrations with Amicon Ultra Centrifugal Filters, Ultracel-30K, biotinylation with BirA enzyme, and FPLC (Superdex 75 column, GE, USA) on AKTA Pure. Finally, the correctly folded pMHC protein was collected and further validated by SDS-PAGE gel electrophoresis (15%) and Flow Cytometry (PE, anti-human β2-microglobulin, BD, Bioscience).

### SPR experiments

SPR experiments were performed with a Biacore 8 K (Cytiva) instrument using Series S sensor SA chip (Cytiva). All experiments were performed in HBS-EP buffer at 25 °C. Throughout the experiment, TCRs flow through the chip as the analytes, and the pMHCs were coated on the chip as ligands. TCRs were finally purified into HBS-EP buffer, and pMHCs were biotinylated as previously described.^27^ For multi-cycle kinetic experiments, the ligands of R4-MHC and L4-MHC were immobilized on the Series S sensor SA chip, at chip densities of roughly 150 response units. TCRs flowed over all flow cells at a flow rate of 30 μL/min, and ${k}_{{{{\rm{on}}}}}$, ${k}_{{{{\rm{off}}}}}$ rates were measured over a series of analyte concentrations ranging from 0 μM to 40 μM for 2C-TCR, and 0 μM to 20 μM for m33-TCR, and 0 μM to 12.5 μM for m67-TCR, respectively. Each analyte injection consisted of a contact time of 60 s followed by a dissociation time of 180 s (2C- and m33-TCRs) or 240 s (m67-TCR). Experimental data were processed in Biacore Insight Evaluation 3.0.12 and were fit to a (1:1) binding model. While calculating the kinetic parameters, ${k}_{{{{\rm{on}}}}}$, ${k}_{{{{\rm{off}}}}}$ and ${R}_{\max }$ were fitted globally.

### Measurements of mouse IL-2 release

To stimulate T hybridomas with RMA-S cells, 1 mL RMA-S cells (1 × 10^6^/mL) were incubated with a serial concentration of different peptides (R4, or L4) respectively in a cell incubator for 6 h. Then the washed 100 μL RMA-S cells (1 × 10^6^/mL) pulsed with cognate peptides were co-cultured with 100 μL T hybridomas (5 × 10^5^/mL) in 96-well plate at 37 °C and 5% CO_2_. After 30 h, the supernatant was harvested and tested with Cytometric Bead Array assay (BD) according to the manufacturer’s manual. As positive control, all T cell lines above were stimulated with plate-coated anti-CD3ε antibody (Clone: 145-2C11, Biolegend) or PMA plus ionomycin. The amount of IL-2 in the supernatant medium was analyzed by Flow Cytometry based on the standard curve. The curve of IL-2 production curve was fitted with GraphPad Prism 10.

### Measurements of NFAT-GFP population

To stimulate MAG-IC3 J76-NFAT-GFP T cells in the absence or presence of CD8, 100 μL T cells (5 × 10^5^/mL) were incubated with a serial concentration of HLA-A*01:01 protein (MAGE-A3, or Titin) respectively in 96-well plate at 37 °C and 5% CO_2_. After 16 h, the NFAT-GFP population of T cells was tested with NovoCyte Advanteon (Agilent) according to the manufacturer’s manual. As positive control, all T cell lines above were stimulated with plate-coated anti-CD3ε antibody (Clone: OKT3, Biolegend). The curve of NFAT-GFP production curve was fitted with GraphPad Prism 10.

### Analysis and calculation of TCR sensitivity and TCR specificity

The sensitivity of each TCR–pMHC pair was calculated as the reciprocal of the lowest antigen concentration required to elicit IL-2 production by T cells, equivalent to approximately 5% of the maximum IL-2 production (P_5_) within a detectable peptide concentration range (i.e., 10^–12^ to 10^–4^ M). The IL-2 response curve was fitted using a quadratic equation:

$$Y=p1\times {x}^{2}+p2\times x+p3,$$

where $Y$ represents the IL-2 production, $x$ represents the antigen concentration, and $p1$, $p2$, and $p3$ are the coefficients. Using this fitting, we identified the antigen concentration corresponding to 5% of the maximum IL-2 production and calculated TCR sensitivity accordingly (Supplementary information, Fig. S1c, d).

TCR specificity was assessed by comparing TCR sensitivity ratios for a cognate antigen (e.g., R4-MHC for 2C-TCR) vs a non-stimulatory antigen (e.g., L4-MHC for 2C-TCR) (Fig. 1g, h).

### Calculation of TCR bond lifetime ratio of antigen A to B and CD8 enhancement power

The TCR bond lifetime ratio $(R)$ for TCR specificity was defined as the ratio of bond lifetimes for TCR binding to an antigen A $({t}_{A})$ vs an antigen B $({t}_{B})$,

$$R=\frac{{t}_{A}}{{t}_{B}},$$

where ${t}_{A}$ represents the bond lifetimes of a TCR binding to the antigen A (e.g., 2C-TCR to R4-MHC, or MAG-IC3-TCR to MAGE-A3), and ${t}_{B}$ represents the bond lifetimes of a TCR binding to antigen B (Figs. 3a–c, left panels, 6e).

Similarly, CD8 enhancement power $(E)$ was defined as the ratio of bond lifetimes for TCR–pMHC interactions in the presence $({t}_{{CD}8+})$ or absence $({t}_{{CD}8-})$ of CD8.

$$E=\frac{{t}_{{CD}8+}}{{t}_{{CD}8-}},$$

where ${t}_{{CD}8+}$ represents the bond lifetimes of TCR–pMHC–CD8 tri-molecular interactions, and ${t}_{{CD}8-}$ represents the bond lifetimes of TCR–pMHC bi-molecular interactions (Figs. 3a–c, 6f);

The SEM for $R$, SEM_*R*_, was calculated using the following error propagation formula:

$${{{{\rm{SEM}}}}}_{R}=R\times \sqrt{{\left(\frac{{{{{\rm{SEM}}}}}_{{t}_{A}}}{{t}_{A}}\right)}^{2}+{\left(\frac{{{{{\rm{SEM}}}}}_{{t}_{B}}}{{t}_{B}}\right)}^{2}},$$

where $\frac{{{{{\rm{SEM}}}}}_{{t}_{A}}}{{t}_{A}}$ and $\frac{{{{{\rm{SEM}}}}}_{{t}_{B}}}{{t}_{B}}$ are the relative errors of ${t}_{A}$ and ${t}_{B}$, respectively. The standard error of the mean (SEM) for ${{{\rm{E}}}}$, ${{{{\rm{SEM}}}}}_{E}$, follows the same equation above.

The bond lifetimes under different forces follow exponential distribution, and the ratios (*R* and *E*) were assumed to follow a normal distribution. By obtaining the mean and standard deviation of *R* and *E*, we performed unpaired student's *t*-tests to compare the differences for TCR specificity (*R*), and used the Mann–Whitney *U*-test to compare the differences of CD8 enhancement power (*E*).

### Preparation of red blood cells (RBCs) and streptavidin (SA) beads

A drop of whole blood was collected from a healthy person’s fingertip into a 1.5 mL tube containing 1 mL C buffer (0.1 M NaHCO_3_, 0.1 M Na_2_CO_3_, pH 8.5, 180 mOsm), adhering to the protocols of the Ethical Review Board of Zhejiang University. Human RBCs were isolated from the whole blood by centrifugation at 2500 rpm, 3 min, and washed twice with 1 mL C buffer. Then the fresh RBCs were biotinylated in 1 mL C buffer containing 1.5 mg/mL Biotin-PEG3500-SGA (JenKem, China) for 30 min at room temperature. Next, the biotinylated RBCs were treated with nystatin (Sigma, Germany) in N2 buffer (265.2 mM KCl, 38.8 mM NaCl, 0.94 mM KH_2_PO_4_, 4.74 mM Na_2_HPO_4_, 27 mM sucrose, pH 7.2, 588 mOsm) at 4 °C for 1 h. Finally, the punched RBCs were washed and resuspended in N2 buffer containing 0.5% bovine serum albumin (BSA, Sigma, Germany).

For streptavidin beads, the 2 μm glass beads (Dry Borosilicate Glass Microspheres, Thermo Fisher Scientific, USA) were sulfhydrylized with mercapto-propyl-trimethoxysilane (Aladdin, China), and then covalently connected with streptavidin-maleimide (SA-MAL, Sigma, Germany) at 4 °C overnight or for 4 h at room temperature. Then, these streptavidin-coated beads were incubated with biotin-pMHC proteins in PBS buffer containing 0.5% BSA (pH 7.2) for 30 min at room temperature for the U-BFP assay.

### Micropipette-based single-cell adhesion frequency assay and 2D effective affinity calculation

The fresh RBCs were biotinylated and then coated with saturated streptavidin, which was further incubated with different concentrations of biotinylated pMHC proteins aspirated by a micropipette. A target T cell, aspirated by an opposed micropipette driven by a nano-scale PZT brake, was brought into contact with the pMHC-coated RBC and retracted within a given time. If the RBC was pulled and deformed, it was recorded as an “adhesion” event. If not, it was recorded as a “no adhesion” event. The experimental cycle was repeated 50 times and tested using 3–5 cell pairs to calculate the adhesion frequency ${P}_{{{{\rm{a}}}}}$. The contact time $({t}_{{{{\rm{c}}}}})$ was varied to control the adhesion frequency within the range of 20%–80%. Finally, the curve of *P*_a_ vs *t*_c_ was obtained based on the adhesion frequency model:

$${P}_{{{{\rm{a}}}}}=1-\exp \{-{m}_{{{{\rm{r}}}}}{m}_{{{{\rm{l}}}}}{A}_{{{{\rm{c}}}}}{K}_{{{{\rm{a}}}}}\left[1-\exp \left({k}_{{{{\rm{off}}}}}{t}_{{{{\rm{c}}}}}\right)\right]\}$$

${m}_{{{{\rm{r}}}}}$ and ${m}_{{{{\rm{l}}}}}$ are the density of the receptor and ligand, respectively, which can be labeled and quantified by fluorescent antibody by Flow Cytometry corresponding to the standard fluorescence intensity bead (BD Biosciences). ${A}_{{{{\rm{c}}}}}$ represents the contact area, and ${K}_{{{{\rm{a}}}}}$ represents the receptor–ligand affinity, and ${k}_{{{{\rm{off}}}}}$ represents the dissociation rates. The 2D affinity of TCR–pMHC interactions was calculated by above equation. In brief, the density of pMHC $({m}_{{{{\rm{r}}}}})$ and TCR $({m}_{{{{\rm{l}}}}})$ was firstly detected by PE anti-human β2-microglobulin (BD, Bioscience) and PE anti-mouse TCRβ antibody, along with BD Quanti-BRITE PE beads for standardization (BD Biosciences). ${A}_{{{{\rm{c}}}}}{K}_{{{{\rm{a}}}}}$ and ${k}_{{{{\rm{off}}}}}$ were then calculated in combination with the ${P}_{{{{\rm{a}}}}}$ values from the micropipette measurements.

### U-BFP assay

The U-BFP setup system with feedback control has been described in detail in the previous paper.^53^ In brief, an SA-bead coated with biotinylated pMHC was attached to the apex of an RBC, which was held by a micropipette on one side. The RBC functioned as an ultra-sensitive biomechanical probe, and its deformation was tracked in real-time by a high-speed camera (Prosilica GE680, Allied Vision, Germany) and transformed into mechanical forces according to its adjustable spring constant (set as 0.3 pN/nm). A target T cell was held by an opposing micropipette and was controlled by the LABVIEW program to contact the ultra-sensitive biomechanical probe for 0.1 s, and then retract until the stretching force reached preset clamp force. The stretching force on the single-molecule bond was precisely clamped until bond rupture, and the duration sustaining clamping force was recorded as the bond lifetime. By collecting bond lifetimes under different force values, the force-regulated dissociation kinetics were dissected. Note that the adhesion frequency of each ligand–receptor pair was controlled to be less than 20% by adjusting the ligand density, ensuring that most recorded adhesive events were mediated by single-molecule bonds. The experiments were performed in a cell chamber filled with complete Dulbecco’s modified Eagle’s medium media (DMEM, Shanghai Basal Media Technology Co., Ltd.) supplemented with 0.5% BSA.

### Measurements of force-dependent bond lifetimes by U-BFP

All force-dependent bond lifetimes were measured by our U-BFP platform. Data in each force-dependent bond lifetime curve were obtained from at least 3 independent experiments, with approximately 300 to 1500 single-molecule bond lifetime measurements collected per curve to ensure the data reliability (Supplementary information, Table S2). In each experiment, we tested multiple preset clamping forces (e.g., 5 pN, 7.5 pN, 10 pN, 12.5 pN, 15 pN, 17.5 pN, 20 pN, etc.) in a random sequence. At each force level, we tested more than 3 different pairs of TCR-expressing target cells and pMHC-coated beads. For each pair, we at least repeatedly tested thousands cycles that included contact, contraction, sustainment, and withdrawal. To ensure most lifetime measurements were mainly from single-molecule events, the adhesion frequency was kept below 20%. Finally, scatter data from all forces were analyzed using our customized LABVIEW program, and the resulting force-dependent mean lifetimes were further analyzed and visualized using GraphPad Prism 10.

### Molecular modeling and MD simulation

The structure of mouse 2C-TCR–R4-H-2K^b^–CD8αβ ternary complex, which is currently unavailable in the PDB database, was generated by assembling the solved structures of 2C-TCR–H-2K^b^ complex (PDB code: 1G6R^52^) with H-2D^d^–CD8αβ complex (PDB code: 3DMM^76^). The assembled structure was then used as the starting model in MD simulations. The ternary complex contains extracellular regions of TCR–pMHC (TCRα Q1–C213, TCRβ E1– C247, SIYR antigen peptide, MHCα G1–W274, and β2m I1–M99), and CD8’s LBD regions (CD8α A4–K121, and CD8β L1–V117). We referred to this model as the R4 complex because super-agonist antigen R4 peptide (SIYRYYGL) is presented in this model. The L4 complex was generated based on the modeled R4 complex structure with the MUTATE plugin in VMD, in which antigen peptide was mutated to L4 (SIYLYYGL). The complex structures of m33- and m67-TCRs with R4-H-2K^b^ were modeled by RoseTTAFold based on the crystal structure of 2C-TCR with R4-H-2K^b^ (PDB code: 1G6R) as the initial template due to the absence of their crystal structures. Then CD8’s LBD region was appended to the modeled m33- and m67-TCRs–R4-H-2K^b^ complex by aligning the H-2K^b^ α_3_ domain of 2C-TCR–R4-H-2K^b^–CD8αβ model built above. Consequently, the models of m33- and m67-TCRs–R4-H-2K^b^–CD8αβ ternary complexes were obtained. The initial structure models of MAG-IC3-TCR–MAGE-A3-HLA-A*01:01 and MAG-IC3-TCR–Titin-HLA-A*01:01 bi-molecular complexes were obtained from PDB database (PDB codes: 5BRZ and 5BS0^15^).

All complex models were rotated to make TCR–pMHC long axis (the line linking C-terminus of MHC and C-terminus of TCR subunits) parallel to the *x*-axis, and then processed with VMD PSFGEN plugin to add hydrogen atoms and other missing atoms. The resulted systems were solvated in rectangular water boxes with TIP3P water model. Na^+^ and Cl^–^ ions were then added to these solvated systems to neutralize the systems and maintain salt concentration at ~150 mM. All systems were first equilibrated with four steps: (1) 10,000 steps energy minimization with the heavy atoms of proteins fixed, followed by 2 ns equilibration simulations under 1 fs timestep with these atoms constrained by 5.0 kcal/mol/Å^2^ spring; (2) 10,000 steps energy minimization with the heavy atoms of proteins fixed, followed by 2 ns equilibration simulations under 1 fs timestep with these atoms constrained by 1.0 kcal/mol/Å^2^ spring; (3) 2 ns equilibration simulations under 1 fs timestep with the heavy atoms of proteins constrained by 0.2 kcal/mol/Å^2^ spring; (4) 10 ns equilibration simulations under 1 fs timestep without constrains. Subsequently, ~100 ns production simulations were carried out with 2 fs time steps under rigid bond algorithms, and the snapshots were saved every 40 ps for further analysis. Two trajectories were generated independently. During the simulations, the temperature of each system was maintained at 310 K with Langevin dynamics and the pressure was controlled at 1 atm with the Nosé-Hoover Langevin piston method. Particle Ewald Mesh summation was used for electrostatic calculation and a 12 Å cutoff with 10 to 12 Å smooth switching was used for short-range non-bonded interactions.

### Cv-SMD simulations

Representative snapshots from the production runs of each system were chosen and extra water molecules were appended to extend the box dimension along with *x*-direction to enable the complex extension in force-loaded cv-SMD simulations. Before applying forces, these models were first equilibrated with a similar strategy as described above. The final configurations were used for the cv-SMD simulations. In each cv-SMD simulation, the C-terminal Cα atoms of TCR and CD8αβ were constrained at their initial positions with a dummy spring (spring constant is 2.0 kcal/mol/Å^2^, ~1400 pN/nm) and the C-terminal Cα atom of MHC was pulled with another dummy spring (spring constant is 0.1 kcal/mol/Å^2^, ~ 70 pN/nm) which moved at ~0.1 nm/ns velocity. The cv-SMD simulations were performed with 1 fs timestep without Langevin temperature and pressure coupling and lasted till the TCR, pMHC and CD8αβ molecules were completely separated, and the snapshots were saved every 20 ps. For each system, a total of 4–5 cv-SMD trajectories were generated for the statistical analyses.

### The analysis of MD trajectories

The conformational change of 2C-TCR–pMHC interface is presented by the line-plane angle (angle α, Supplementary information, Fig. S6a–c) between the α-helix peptide of H-2K^b^ (E58–Y84), which is expressed by a vector connecting the centroids of E58–G69 and S73–Y84 backbone atoms, and CDR regions of 2C-TCR, which is expressed by a plane defined by the three centroids of CDR1α–CDR2α, CDR3α–CDR2β, and CDR1β–CDR3β. The conformational change of β2m–MHC α_3_ interface is presented by the vector angle (angle β, Supplementary information Fig. S6d, e) between β2m, which is expressed by a vector connecting centroids of Q6, I7, Q29, F30, F62, Y63 and S11, R12, L23, N24, T68, E69 backbone atoms, and MHC α_3_ domain, which is expressed by a vector connecting centroid of G207, F208, P235, A236, F241, Q242 and R202, C203, M228, G229, S246, V247 backbone atoms. The conformational change of CD8β–pMHC interface is presented by the vector angle (angle γ, Supplementary information, Fig. S6f, h) between CD8β, which is expressed by a vector connecting centroids of L1-Q3/S19-E21 and L9-Q11/H14-A16 backbone atoms, and the pMHC, which is expressed by a vector connecting centroids of MHC α_1_α_2_ and α_3_ domains. The contact areas between CD8β and MHC and between Ile2 of CD8β and the hydrophobic patch of H-2K^b^ (F8, I98, Y113) and β2m (W60) are calculated individually. The distance threshold of H-bond was set to 3.5 Å between the donor and acceptor atoms, and angle cutoff was set to 50°. Three different conformational states of TCR–pMHC–CD8 complex are defined for understanding their conformational characters conveniently. The first state, referred to as the *I*_0_ state, is defined as the structure snapshots of the first 5 ns of SMD trajectories; the second state, referred to as *I*_1_ state, is defined as the structure snapshots after the occurrence of conformational changes between 2C-TCR and H-2K^b^ and before the occurrence of conformational changes between CD8 and H-2K^b^ interface in the SMD trajectories; and the third state, referred to as *I*_2_ state, is defined as the structure snapshots after conformational changes of CD8–H-2K^b^ interface happened and before 2C-TCR and H-2K^b^ was separated. For the MAG-IC3-TCR–HLA-A*01:01 systems, the definition of angles α and β are similar with those in the 2C-TCR–H-2K^b^ systems. All simulations were performed with NAMD2 software using CHARMM36m force field with the CMAP correction.^77,78^ The system preparations and trajectory analyses were conducted with VMD.^79^ Illustrations of the representative frames shown in the Figures and the Supplementary Figures were rendered by UCSF ChimeraX.

### Mathematical model

#### Bi-molecular model for TCR–pMHC interaction

The bi-molecular model was constructed with a sequence of reaction steps (Supplementary information, Fig. S9a), including one initial step where pMHC firstly encounters and associates with TCR in proximity, and multiple intermediate steps where the engaged TCR–pMHC complex (*B*_i_) reacts forward to be further phosphorylated (with the reaction rate ${k}_{{{{\rm{p}}}}}$) or backward to dissociate. The dissociation of TCR–pMHC is characterized by the force-dependent off-rates $({k}_{{{{\rm{off}}}}})$ obtained from the aforementioned U-BFP assay. Taken into account the intracellular conformational changes of TCR through phosphorylation reactions, both a fast-rebinding step (with the reaction rate ${k}_{{{{\rm{on}}}}-{{{\rm{rebinding}}}}}$) and a reacting forward step (with the reaction rate ${k}_{{{{\rm{p}}}}}$) are introduced for the TCR–pMHC complex that has just disassociated but still within close contact. Additionally, the dissociated intermediates can also fully fall apart and return to the initial unmodified state $({U}_{0})$ with a constant reaction rate (*μ*). The final step represents the engaged TCR in a fully phosphorylated state $({B}_{{{{\rm{s}}}}})$ that is able to trigger downstream signals. The probability of TCR retained in ${B}_{{{{\rm{s}}}}}$ state is defined as the recognition signal triggered by the TCR–pMHC pair. In the simulation, the probability with a value less than 0.5 is considered as a non-functional signal. The bi-molecular model was implemented with the ordinary differential equations (ODEs) as follows:

Step 0:

$$\frac{\partial {B}_{0}}{\partial t}=-\left({k}_{{{{\rm{p}}}}}+{k}_{{{{\rm{off}}}}}\right)\times {B}_{0}+{k}_{{{{\rm{on}}}}-{{{\rm{initial}}}}}\times {U}_{0}$$

$$\frac{\partial {U}_{0}}{\partial t}=-{k}_{{{{\rm{on}}}}-{{{\rm{initial}}}}}\times{U}_{0}+{k}_{{{{\rm{off}}}}}\times{B}_{0}+\mu\times{\sum }_{i=1}^{s-1}{U}_{i}$$

Step 1:

$$\normalsize \normalsize \normalsize \normalsize \normalsize \normalsize \frac{\partial {B}_{1}}{\partial t}=-\left({k}_{{{{\rm{p}}}}}+{k}_{{{{\rm{off}}}}}\right)\times{B}_{1}+{k}_{{{{\rm{on}}}}-{{{\rm{rebinding}}}}}\times{U}_{1}+{{k}_{{\rm{p}}}}\times{B}_{0}$$

$$\frac{\partial {U}_{1}}{\partial t}=-\left({k}_{{{{\rm{p}}}}}+{k}_{{{{\rm{on}}}}-{{{\rm{rebinding}}}}}+\mu \right)\times{U}_{1}+{k}_{{{{\rm{off}}}}}\times{B}_{1}$$

Step 2~s–2:

$$\frac{\partial {B}_{i}}{\partial t}=-\left({k}_{{{{\rm{p}}}}}+{k}_{{{{\rm{off}}}}}\right)\times{B}_{i}+{k}_{{{{\rm{on}}}}-{{{\rm{rebinding}}}}}\times {U}_{i}+{k}_{{{{\rm{p}}}}}\times{B}_{i-1}$$

$$\frac{\partial {U}_{i}}{\partial t}=-\left({k}_{{{{\rm{p}}}}}+{k}_{{{{\rm{on}}}}-{{{\rm{rebinding}}}}}+\mu \right)\times{U}_{i}+{k}_{{{{\rm{off}}}}}\times{B}_{i}+{k}_{{{{\rm{p}}}}}\times{U}_{i-1}$$

Step s-1:

$$\frac{\partial {B}_{s-1}}{\partial t}=-\left({k}_{{{{\rm{p}}}}}+{k}_{{off}}\right)\times{B}_{s-1}+{k}_{{{{\rm{on}}}}-{{{\rm{rebinding}}}}}\times{U}_{s-1}+{k}_{{{{\rm{p}}}}}\times{B}_{s-2}$$

$$\frac{\partial {U}_{s-1}}{\partial t}=-\left({k}_{{{{\rm{on}}}}-{{{\rm{rebinding}}}}}+\mu \right)\times{U}_{s-1}+{k}_{{{{\rm{off}}}}}\times{B}_{s-1}+{k}_{{{{\rm{p}}}}}\times{U}_{s-2}$$

Step s:

$$\frac{\partial {B}_{s}}{\partial t}={k}_{{{{\rm{p}}}}}\times{B}_{s-1}$$

In these equations, ${B}_{i}$ represents the engaged TCR–pMHC state and ${U}_{i}$ represents the state that pMHC dissociates with TCR but stays still in proximity. The initial binding of pMHC with TCR is characterized by the initial on-rates $({k}_{{{{\rm{on}}}}-{{{\rm{initial}}}}})$ which were measured in micropipette assay (Supplementary information, Table S1). Once the complex ${B}_{i}$ is formed, it can either react into a more phosphorylated state *B*_*i*+1_ or dissociate to the proximity state ${U}_{i}$ at off-rates $({k}_{{{{\rm{off}}}}})$. For the variables in ${U}_{i}$ states, they can either react into a more phosphorylated state ${U}_{i+1}$, or reform the bound complex $({B}_{i})$ with a faster rebinding rate, or directly return to the unmodified state $({U}_{0})$ at proofreading rate $\mu$. In the simulation, the number of intermediate steps was set as 4, and the values of the parameter ${k}_{{{{\rm{p}}}}}$ and $\mu$ were set as 3 and 1 s^−1^, respectively. The value of ${k}_{{{{\rm{off}}}}}$ was calculated as the reciprocal of the bond lifetime in the medium force regime for each TCR pair. If the lifetime in the medium force regime was unavailable, the corresponding peak bond lifetime was used instead. The value of ${k}_{{{{\rm{on}}}}-{{{\rm{rebinding}}}}}$ was set as a thousand times of ${k}_{{{{\rm{on}}}}-{{{\rm{initial}}}}}$ for each TCR pair.

### Tri-molecular model for TCR–pMHC–CD8 interaction

The tri-molecular model for TCR and pMHC interaction with the presence of CD8 was constructed in a similar way as the bi-molecular model. It used the same model structure and the same parameter sets, except for the forward reaction rate ${k}_{{{{\rm{p}}}}}$ and the off-rate ${k}_{{{{\rm{off}}}}}$ for TCR–pMHC–CD8 complex. Based on SMD simulations and the significant CD8αβ–Lck occupancy reported in the recent study,^60^ we hypothesized that CD8 might partially modulate TCR–pMHC recognition by affecting the availability of effective Lck capable of phosphorylating TCR intracellularly, leading to the accelerated phosphorylation of the engaged TCR complex.^80^ Thus, the value of ${k}_{{\rm{p}}}$ in the tri-molecular model should be no longer constant, but varying according to the function of CD8. Therefore, we proposed the dynamic curve of ${k}_{{{{\rm{p}}}}}$ to have a similar shape as the curve for CD8 enhancement power (Fig. 6f; Supplementary information, Fig. S9d, e), and evaluated its effectiveness by comparing the predicted signals with the experimentally observed functions for the six TCR pairs in 2C-TCR system. The value of ${k}_{{{{\rm{off}}}}}$ was calculated by the reciprocal of the tri-molecular bond lifetime. To use the bi-molecular lifetime as model input, we implemented curve fitting by using the bi- and tri-molecular lifetimes measured for the six TCR pairs in 2C-TCR system, which was then used to estimate the corresponding tri-molecular lifetime for a given bi-molecule lifetime (Supplementary information, Fig. S9e). The value of ${k}_{{{{\rm{on}}}}-{{{\rm{initial}}}}}$ was calculated as $\frac{{A}_{{{{\rm{c}}}}}{K}_{{{{\rm{a}}}}}}{{A}_{{{{\rm{c}}}}}} \times {k}_{{{{\rm{off}}}}},{A}_{{{{\rm{c}}}}}=\pi \times{0.75}^{2}$ in situ binding affinities $({A}_{{{{\rm{c}}}}}{K}_{{{{\rm{a}}}}})$. The values of other parameters were kept the same as the bi-molecular model.

### Statistical analysis

Data comparisons in this study were conducted using paired, or unpaired *t*-tests, or the Mann–Whitney test, depending on the experimental design. All analyses were performed using GraphPad Prism 10. Statistical significance was defined as follows: ns not significant, *P < 0.05, **P < 0.01, ***P < 0.001, ****P < 0.0001. Correlation analyses were evaluated using the Pearson correlation coefficient (*r*).

## Supplementary information

Fig. S1

Fig. S2

Fig. S3

Fig. S4

Fig. S5

Fig. S6

Fig. S7

Fig. S8

Fig. S9

Fig. S10

Fig. S11

Table S1

Table S2

Table S3

Table S4

Table S5

Table S6

Table S7

Table S8

## Data Availability

All data are available in the main text or the Supplementary Materials.
