# Supplementary material for: TCR catch bonds nonlinearly control CD8 cooperation to shape T cell specificity
Source: Cell Res. 2025 Feb 27;35(4):265–83. doi: 10.1038/s41422-025-01077-9 (PMC11958657; doi:10.1038/s41422-025-01077-9)
Supplement: Supplementary file 1 — Fig. S1 [file 41422_2025_1077_MOESM1_ESM.pdf]

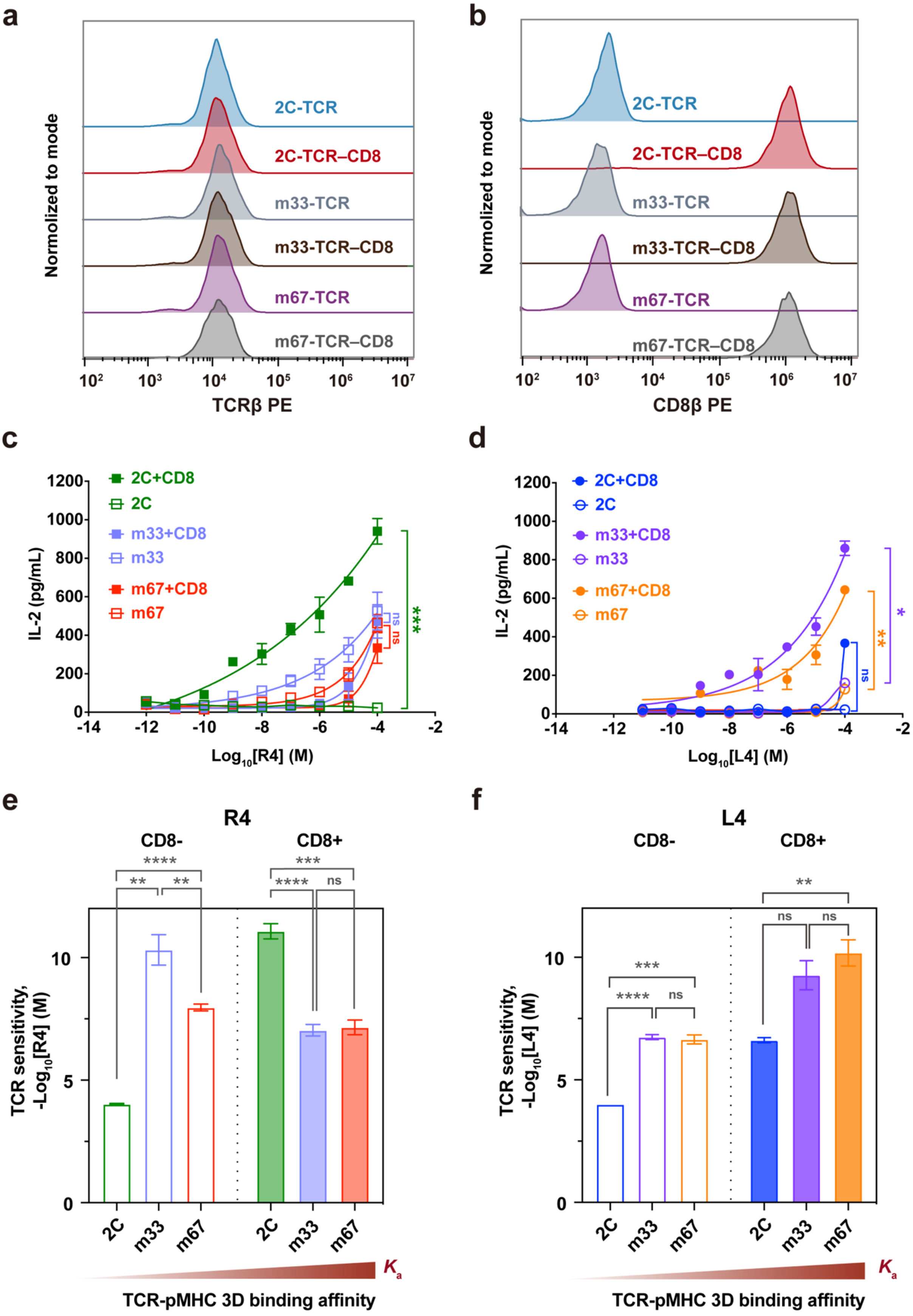
a
Normolized to mode
2C-TCR
2C-TCR–CD8
m33-TCR
m33-TCR–CD8
m67-TCR
m67-TCR–CD8
TCRβ PE
b
Normolized to mode
2C-TCR
2C-TCR–CD8
m33-TCR
m33-TCR–CD8
m67-TCR
m67-TCR–CD8
CD8β PE
c
2C+CD8
2C
m33+CD8
m33
m67+CD8
m67
IL-2 (pg/mL)
Log10[R4] (M)
d
2C+CD8
2C
m33+CD8
m33
m67+CD8
m67
IL-2 (pg/mL)
Log10[L4] (M)
e
R4
CD8-
CD8+
TCR sensitivity, -Log10[R4] (M)
2C
m33
m67
TCR-pMHC 3D binding affinity
f
L4
CD8-
CD8+
TCR sensitivity, -Log10[L4] (M)
2C
m33
m67
TCR-pMHC 3D binding affinity

Supplementary information, Fig. S1 Strengthening the 3D affinity of TCR–pMHC binding through mutagenesis on TCR CDR3α modulates CD8 enhancement power for TCR sensitivity. **a, b** The staining of 2C-, m33-, and m67-TCRs molecules expressed on 58α-β- hybridomas cells using PE anti-mouse TCRβ antibody (**a**), and the expression of CD8 molecules on 2C, m33, m67 hybridomas cells using PE anti-mouse CD8β antibody (clone: eBioH35-17.2) (**b**). **c, d** IL-2 production of 2C, m33 and m67 hybridoma T cells expressing CD8 or not, when stimulated by RMA-S cells pulsed with different concentrations of R4 (**c**) or L4 (**d**) peptide. The statistical analyses were conducted using the Mann-Whitney test. Notably, the data from Fig. 1c–f were replotted in Fig. S1c, d to compare CD8's contribution across different TCR–pMHC pairs. Data of Fig. 1c and Fig. 1d are from the same experiment but separately presented for better comparisons among 2C, m33, m67 hybridomas cells**.** Data of Fig. 1e and Fig. 1f are also from the same experiment. **e, f** TCR sensitivity of 2C, m33, and m67 hybridomas cells recognizing R4 (**e**) or L4 (**f**) peptide in the presence or absence of CD8; the statistical analyses were performed by unpaired *t*-tests. In Fig. S1c–f, error bars are ± SEMs (n $\geq$ 3), and the statistical significance was indicated as follows: *$P < 0.05$, **$P < 0.01$, ***$P < 0.005$, ****$P < 0.0001$.
