# Supplementary material for: TCR catch bonds nonlinearly control CD8 cooperation to shape T cell specificity
Source: Cell Res. 2025 Feb 27;35(4):265–83. doi: 10.1038/s41422-025-01077-9 (PMC11958657; doi:10.1038/s41422-025-01077-9)
Supplement: Supplementary file 2 — Fig. S2 [file 41422_2025_1077_MOESM2_ESM.pdf]

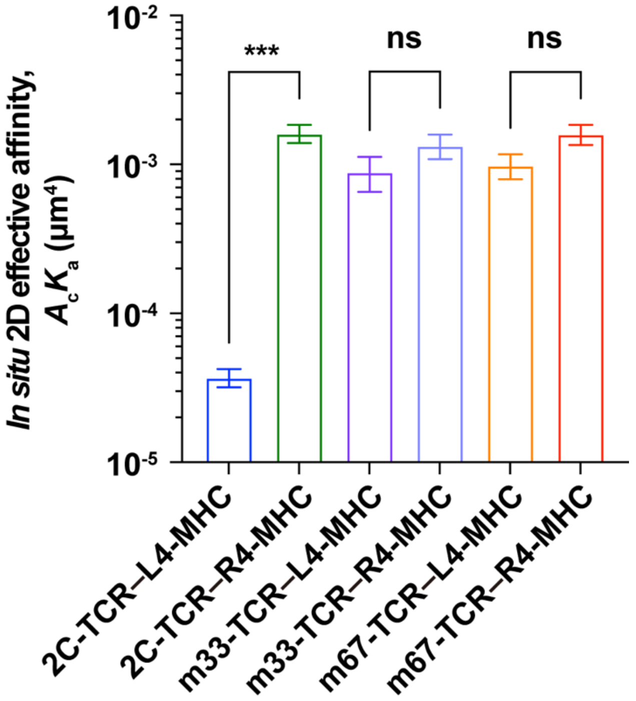

Supplementary information, Fig. S2 In situ 2D effective affinity of 2C-, m33-, or m67-TCRs interacting with R4- or L4-MHCs. The data is presented as mean ± SEMs (n > 4) and summarized in Supplementary information, Table S1. The statistical analyses were performed by unpaired *t*-tests; the statistical significance was indicated as follows: $*P < 0.05$, $**P < 0.01$, $***P < 0.005$.
