# Supplementary material for: TCR catch bonds nonlinearly control CD8 cooperation to shape T cell specificity
Source: Cell Res. 2025 Feb 27;35(4):265–83. doi: 10.1038/s41422-025-01077-9 (PMC11958657; doi:10.1038/s41422-025-01077-9)
Supplement: Supplementary file 3 — Fig. S3 [file 41422_2025_1077_MOESM3_ESM.pdf]

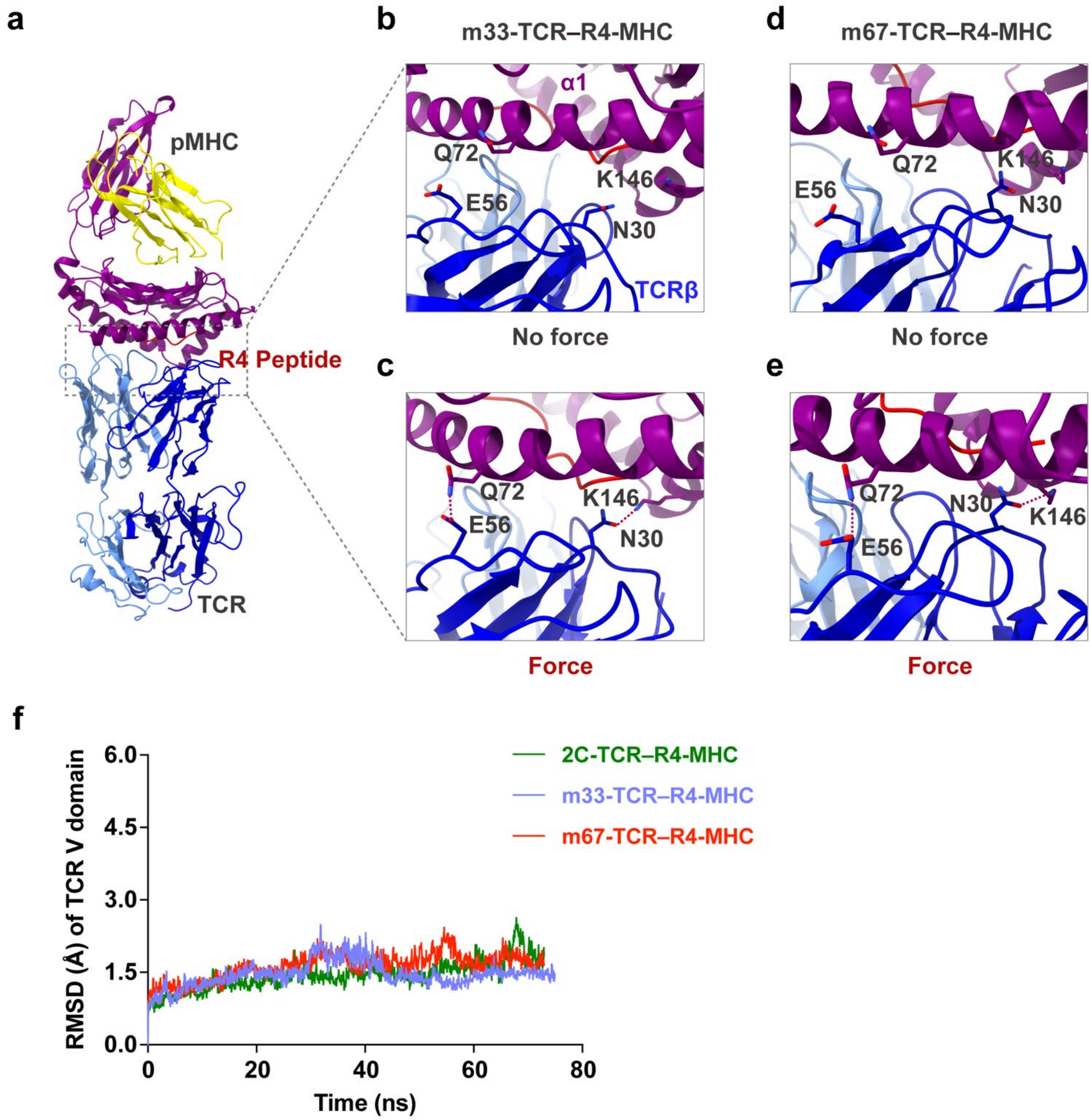

Supplementary information, Fig. S3 Mechanical force induces additional H-bonds formation of MHC binding with TCRβ chain, while m33- and m67-TCRs do not undergo significant structural changes.

a–**e** The representative cv-SMD snapshots of m33-, or m67-TCRs in complex with R4-MHC under force and zoomed-in contact interface under without force (**b, d**) or under force (**c, e**) between TCRβ chains and MHC. Shown are representative snapshots of H-bond formations between N30 residue on the m33-TCRβ (**b, c**) or m67-TCRβ (**d, e**) and MHC-K146, as well as between E56 residue on the m33-TCRβ (**b, c**) or m67-TCRβ (**d, e**) and MHC-Q72 under force or without force. H-bonds are indicated as dashed red lines. **f** The RMSD values of TCR V domain during the dynamic dissociation pathway of m33-TCR–R4-MHC or m67-TCR–R4-MHC in cv-SMD simulations.
