# Supplementary material for: TCR catch bonds nonlinearly control CD8 cooperation to shape T cell specificity
Source: Cell Res. 2025 Feb 27;35(4):265–83. doi: 10.1038/s41422-025-01077-9 (PMC11958657; doi:10.1038/s41422-025-01077-9)
Supplement: Supplementary file 4 — Fig. S4 [file 41422_2025_1077_MOESM4_ESM.pdf]

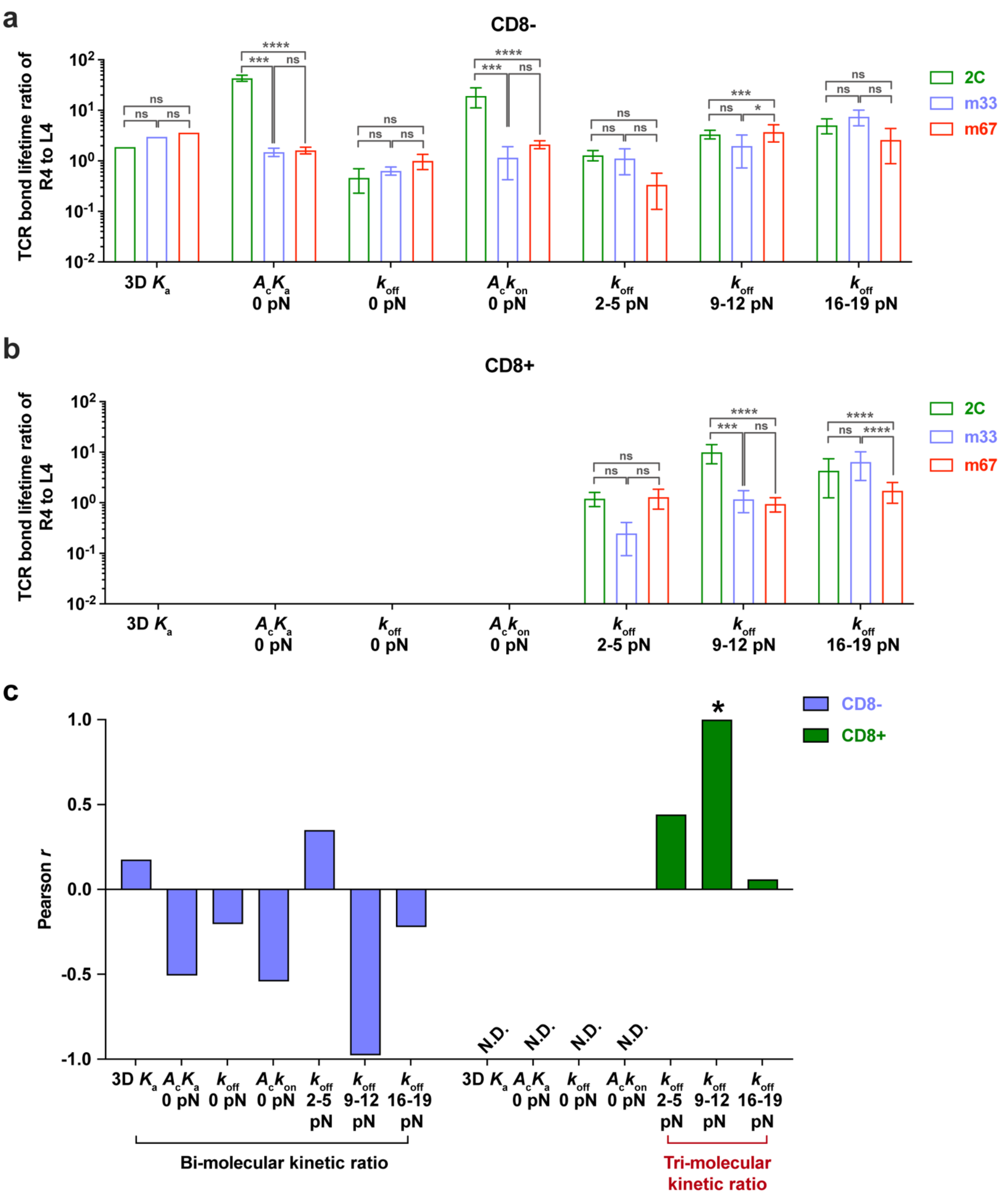

Supplementary information, Fig. S4 Correlation analysis of TCR specificity with different TCR–pMHC binding kinetics in the absence or presence of CD8.

a, b The ratio of bi-molecular TCR–pMHC binding (**a**) or tri-molecular TCR–pMHC–CD8 binding (**b**), including 3D binding affinity (K_a), 2D effective affinity ($A_c K_a$), or zero-force off-rates (k_{off}), or zero-force on-rates ($A_c k_{on}$), or bond lifetimes in the low (2–5 pN), medium (9–12 pN), or high (16–

19 pN) regime. The statistical analyses were performed using unpaired *t*-tests. The TCR bond lifetime ratios in the presence and absence of CD8 from Fig. 3a–c left panels were replotted in Fig. S5a, b for comparison. **c** Comparison of the Pearson correlation coefficient (*r*) between TCR bond lifetime ratio of R4 to L4 in the absence or presence of CD8 and TCR specificity; the statistical significance was indicated as follows: $*P < 0.05$, $**P < 0.01$, $***P < 0.005$, $****P < 0.0001$. N.D. indicates not detected. Error bars are ± SEMs.
