# Supplementary material for: TCR catch bonds nonlinearly control CD8 cooperation to shape T cell specificity
Source: Cell Res. 2025 Feb 27;35(4):265–83. doi: 10.1038/s41422-025-01077-9 (PMC11958657; doi:10.1038/s41422-025-01077-9)
Supplement: Supplementary file 5 — Fig. S5 [file 41422_2025_1077_MOESM5_ESM.pdf]

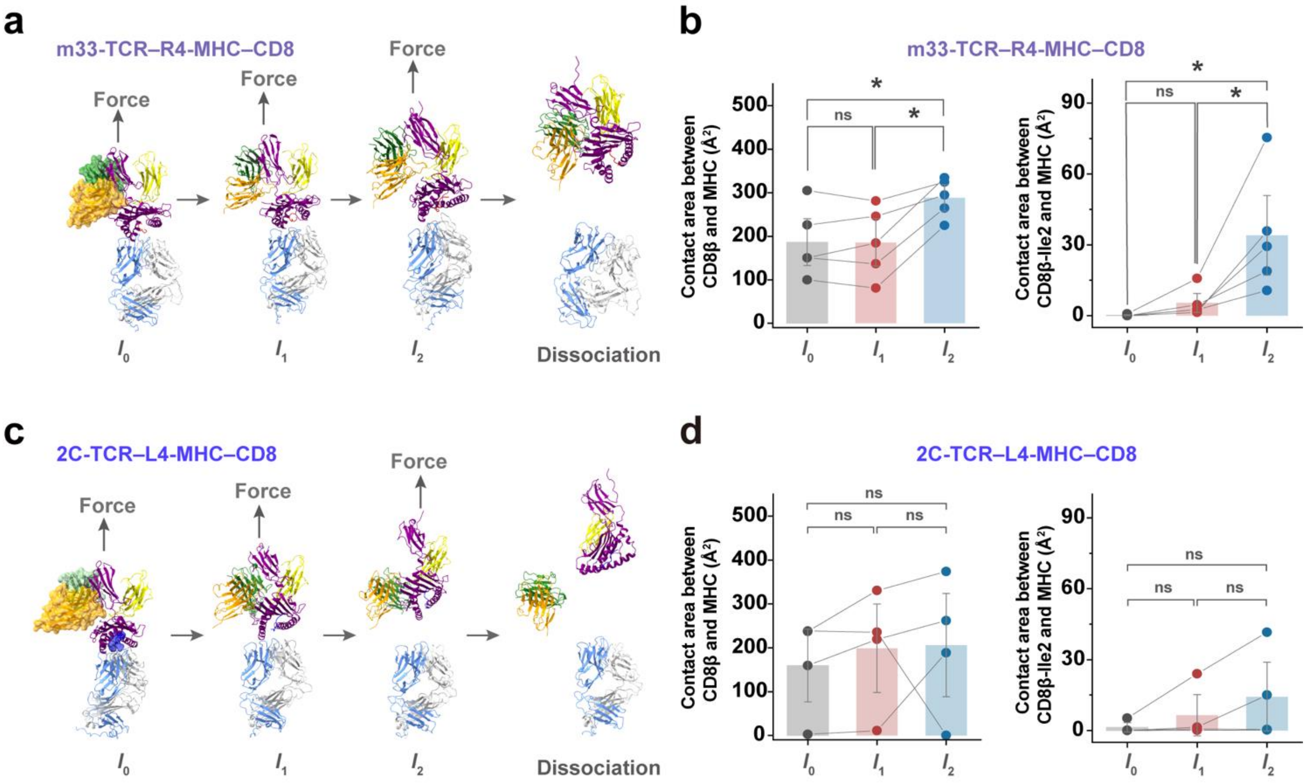

Supplementary information, Fig. S5 TCR–pMHC binding with weak or ultra-strong bond lifetime selectively constrains CD8 rotation.

a–**d** (**a, c**) Sequential snapshots of cv-SMD simulations depicting the m33-TCR–R4-MHC–CD8αβ (**a**) or 2C-TCR–L4-MHC–CD8αβ (**c**) complex under mechanical force pulling; (**b, d**) The contact area between CD8β or its Ile2 residue and MHC under mechanical force loading in cv-SMD simulations. Error bars are ± SEMs. The statistical analyses were performed by unpaired *t*-tests; the statistical significance was indicated as follows: *$P < 0.05$, **$P < 0.01$, ***$P < 0.005$, ****$P < 0.0001$.
