# Supplementary material for: TCR catch bonds nonlinearly control CD8 cooperation to shape T cell specificity
Source: Cell Res. 2025 Feb 27;35(4):265–83. doi: 10.1038/s41422-025-01077-9 (PMC11958657; doi:10.1038/s41422-025-01077-9)
Supplement: Supplementary file 6 — Fig. S6 [file 41422_2025_1077_MOESM6_ESM.pdf]

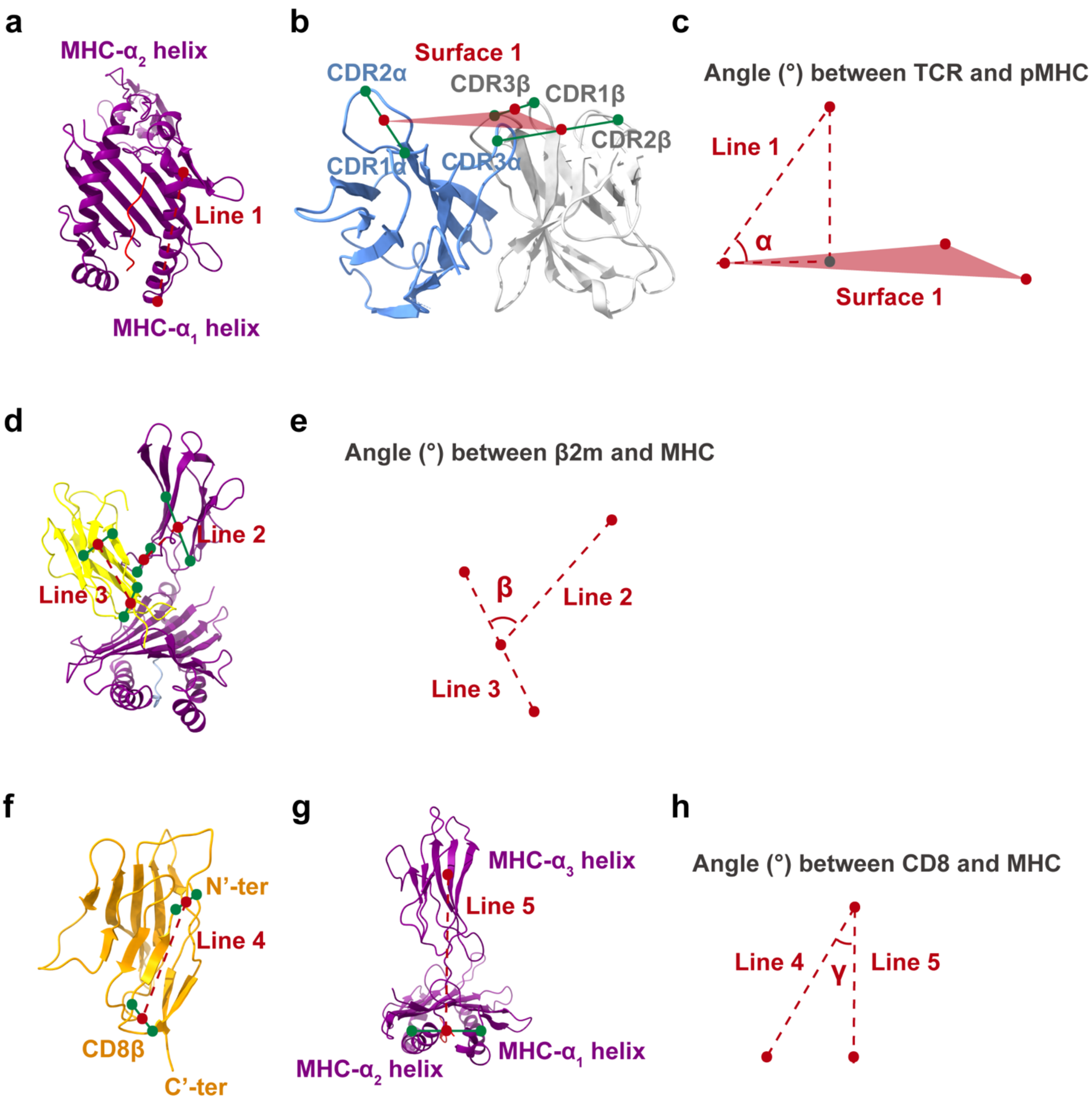

Supplementary information, Fig. S6 The vector-based analysis of the TCR–pMHC–CD8 ternary complex reveals the relative conformational changes among the pMHC, TCR and CD8 molecules. **a**–**h** A simplified diagram illustrating the relationship between H-2K^b and the variable domain of TCRαβ protein (**a**, **b**), or between β2m and MHC (**d**, **e**), or between CD8β and H-2K^b (**f**–**h**) depicting the connecting centroids, and the angle between line 1 and surface 1, with the designated angle (α) labelled (**c**); or the angle between line 2 and line 3, with the designated angle (β) labelled (**e**); or the angle between line 4 and line 5, with the designated angles (γ) labelled (**h**).
