# Supplementary material for: TCR catch bonds nonlinearly control CD8 cooperation to shape T cell specificity
Source: Cell Res. 2025 Feb 27;35(4):265–83. doi: 10.1038/s41422-025-01077-9 (PMC11958657; doi:10.1038/s41422-025-01077-9)
Supplement: Supplementary file 8 — Fig. S8 [file 41422_2025_1077_MOESM8_ESM.pdf]

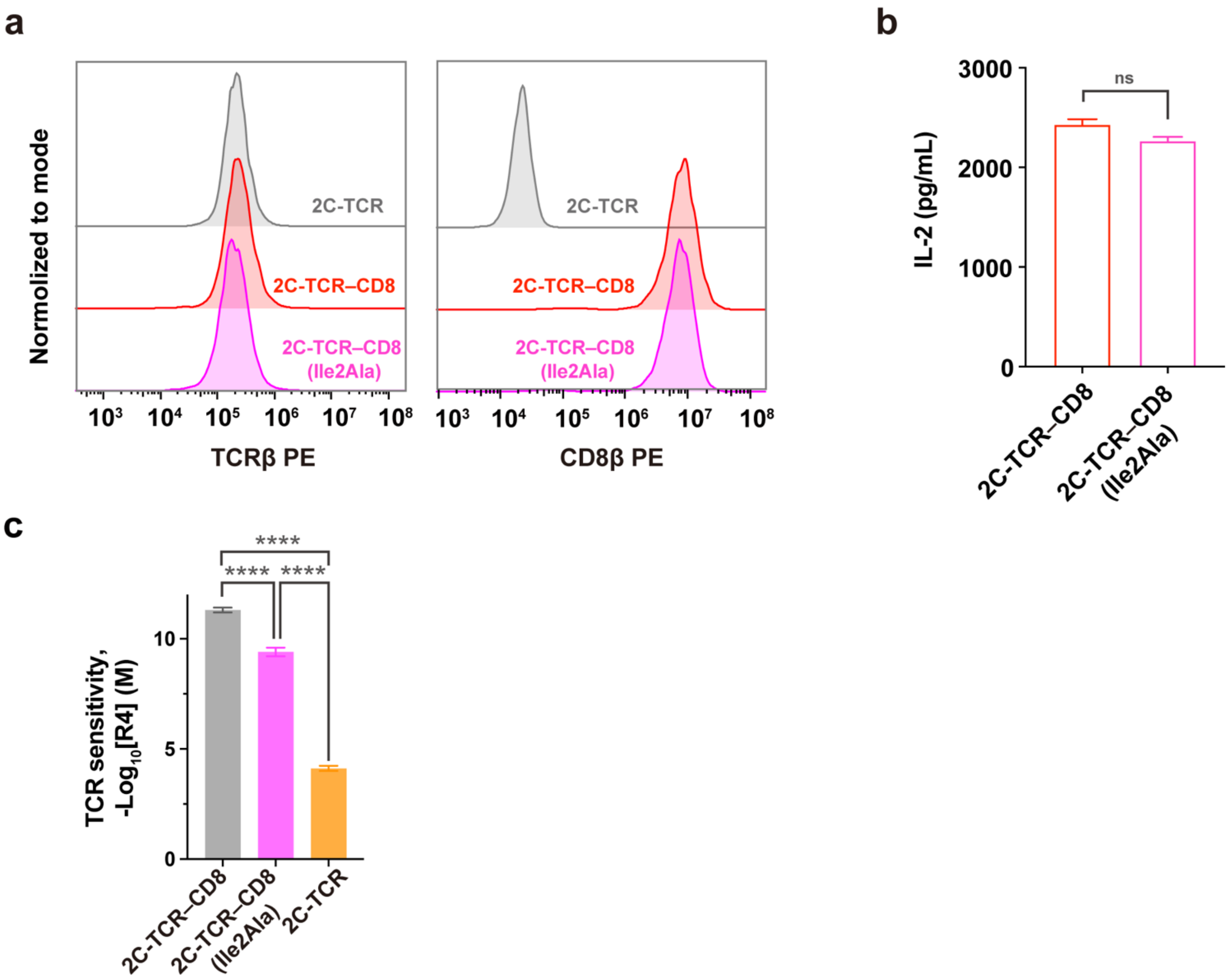

Supplementary information, Fig. S8 Blocking force-enhanced interactions at the CD8–MHC interface impairs the TCR sensitivity.

a The staining of CD8 (Ile2Ala) molecules expressed on 2C hybridoma cells using PE anti-mouse CD8β antibody. **b** IL-2 release from 2C hybridoma cells expressing CD8 or CD8 (Ile2Ala) upon stimulation with plate-coated 5 μg/mL anti-mouse CD3ε antibody (145-2C11). **c** TCR sensitivity of 2C-TCR binding to R4-MHC in the presence of CD8 or CD8 (Ile2Ala), or in the absence of CD8. Error bars are ± SEMs. The statistical analyses in panels (**b**, **c**) were performed by unpaired *t*-tests; the statistical significance was indicated as follows: *$P < 0.05$, **$P < 0.01$, ***$P < 0.005$, ****$P < 0.0001$.
