# Supplementary material for: TCR catch bonds nonlinearly control CD8 cooperation to shape T cell specificity
Source: Cell Res. 2025 Feb 27;35(4):265–83. doi: 10.1038/s41422-025-01077-9 (PMC11958657; doi:10.1038/s41422-025-01077-9)
Supplement: Supplementary file 9 — Fig. S9 [file 41422_2025_1077_MOESM9_ESM.pdf]

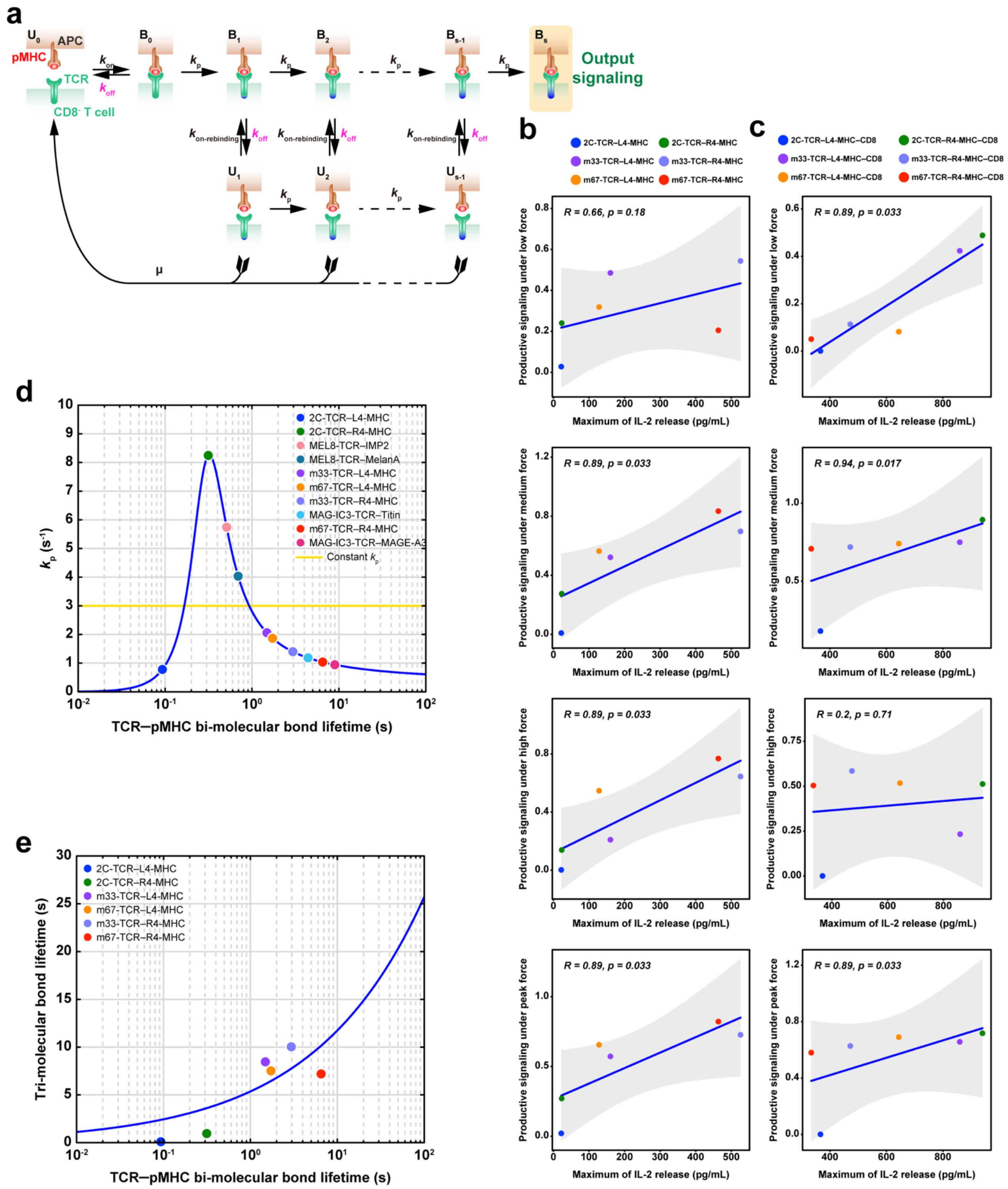

Supplementary information, Fig. S9 The signal probability induced in the medium force regime exhibits a positive correlation with IL-2 release.

a Schematic diagram of the force-dependent kinetic proofreading model of TCR triggering in the absence of CD8. **b, c** Correlation between the probabilities of productive signaling in the low, or medium, or high, or peak force regimes and the maximum release of IL-2 under the peptide

concentration at a magnitude of 10^{-4} M in the absence (**b**) or presence (**c**) of CD8. **d** The forward rate (k_{p}) of force-dependent KPR model, in the presence of CD8, fluctuates in accordance with the bond lifetimes of TCR–pMHC bi-molecules. **e** The tri-molecular bond lifetimes in the medium force regime varies with the bond lifetimes of TCR–pMHC bi-molecular complexes in the medium force regime.
