# Supplementary material for: TCR catch bonds nonlinearly control CD8 cooperation to shape T cell specificity
Source: Cell Res. 2025 Feb 27;35(4):265–83. doi: 10.1038/s41422-025-01077-9 (PMC11958657; doi:10.1038/s41422-025-01077-9)
Supplement: Supplementary file 10 — Fig. S10 [file 41422_2025_1077_MOESM10_ESM.pdf]

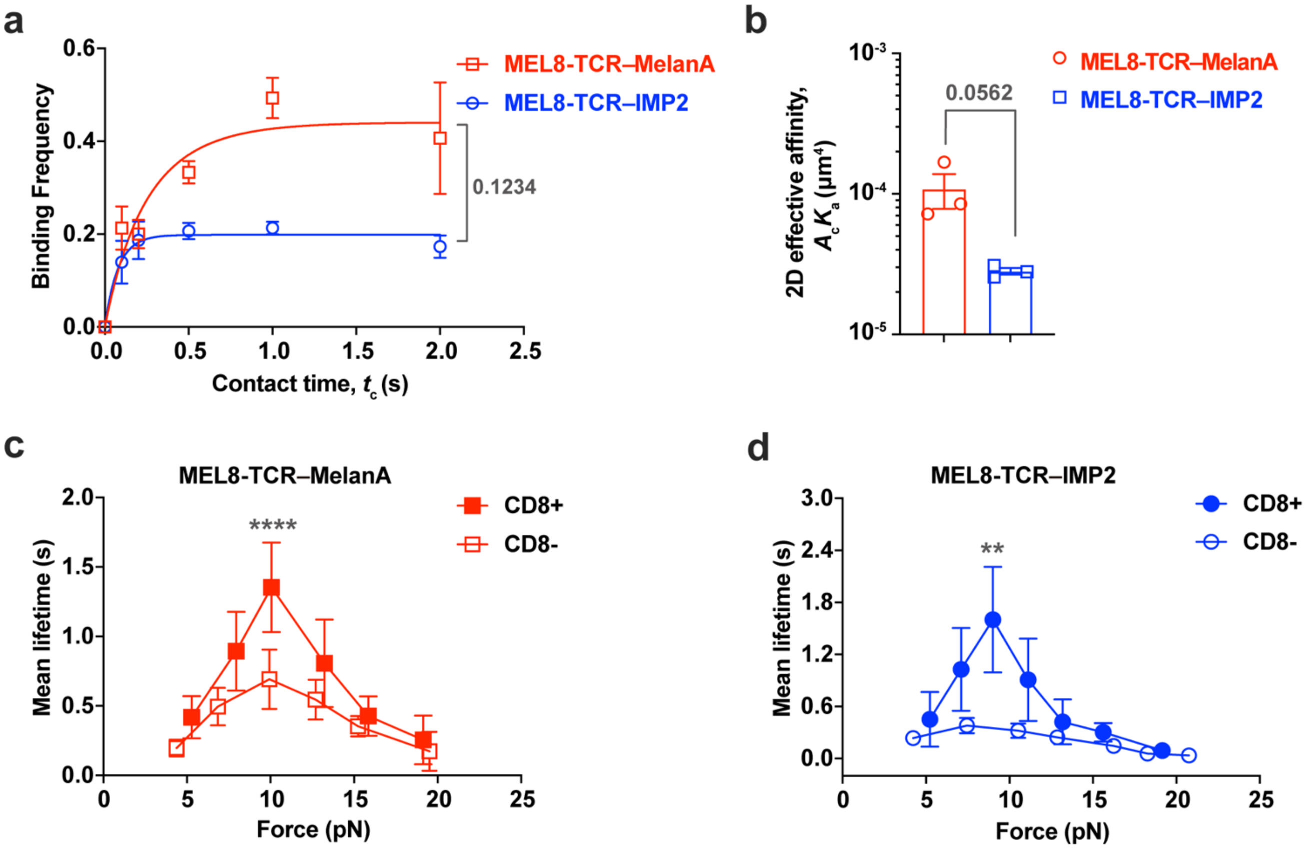

Supplementary information, Fig. S10 The binding kinetics of naturally MEL8-TCR with two tumor associated antigens, MelanA and IMP2.

a, b In situ 2D binding frequency (**a**) and 2D effective affinity (**b**) of MEL8-TCR interacting with MelanA-HLA-A*02:01 and IMP2-HLA-A*02:01). The data are presented as mean ± SEMs (n > 4) and summarized in Supplementary information, Table S1. The statistical analysis in panel **a** was performed using the Mann-Whitney test, and in panel **b** was performed using unpaired *t*-tests. **c, d** The force-dependent bond lifetimes curves of MEL8-TCR binding to MelanA-HLA-A*02:01 (**c**) and IMP2-HLA-A*02:01 (**d**) in the absence (open-symbol plots) or presence (closed-symbol plots) of CD8. The number of bond lifetimes for different TCR–pMHC or TCR–pMHC–CD8 pairs is summarized in Supplementary information, Table S2. All binned data points of force curves are presented as mean ± SEMs and summarized in Supplementary information, Tables S3 and S4. The statistical analyses of peak bond lifetime in panels (**c**, **d**) were performed by the Mann-Whitney test; the statistical significance was indicated as follows: $*P < 0.05$, $**P < 0.01$, $***P < 0.005$.
