# Supplementary material for: TCR catch bonds nonlinearly control CD8 cooperation to shape T cell specificity
Source: Cell Res. 2025 Feb 27;35(4):265–83. doi: 10.1038/s41422-025-01077-9 (PMC11958657; doi:10.1038/s41422-025-01077-9)
Supplement: Supplementary file 11 — Fig. S11 [file 41422_2025_1077_MOESM11_ESM.pdf]

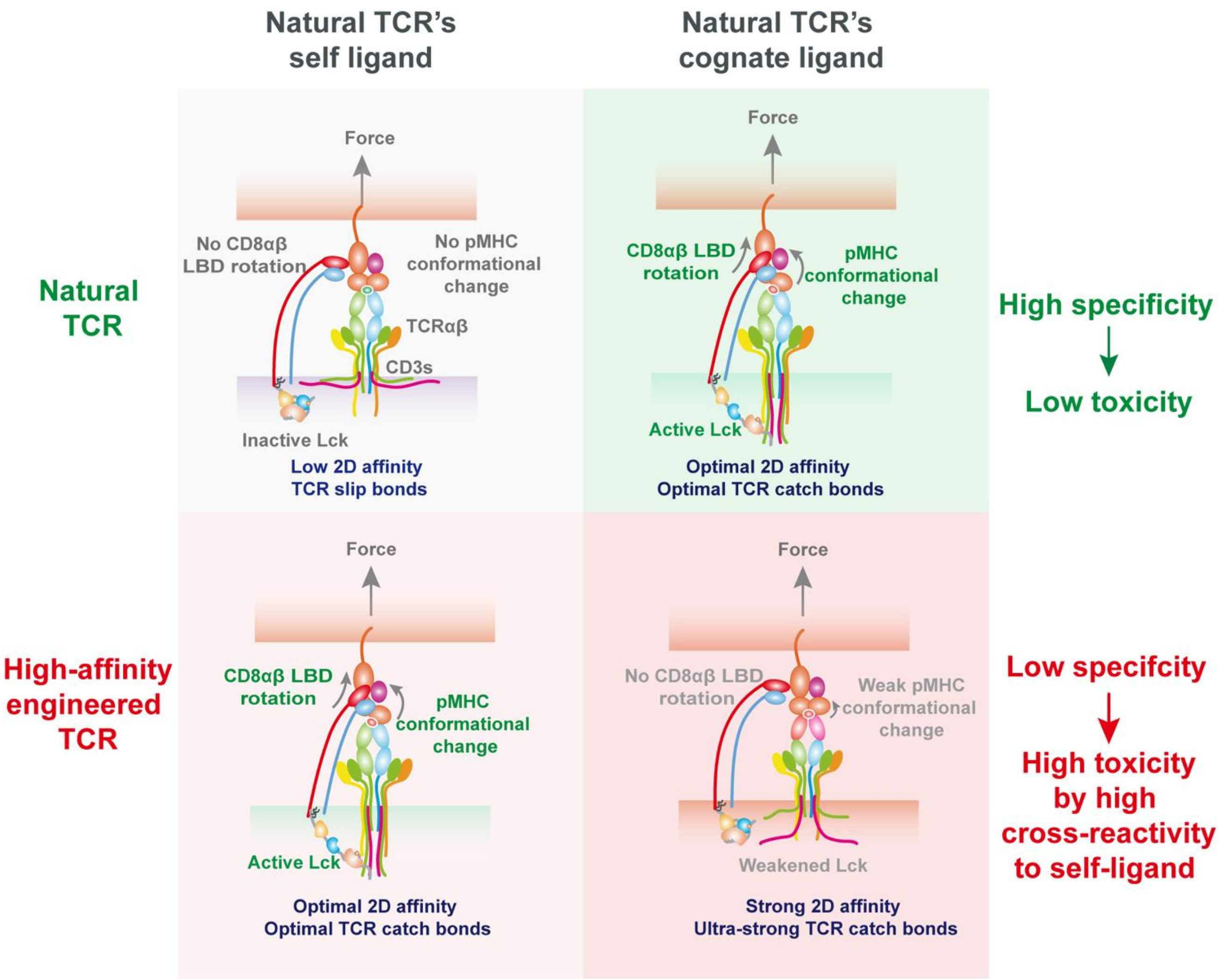

Supplementary information, Fig. S11 The mechano-biochemical model of TCR catch bonds dynamically shape T cell antigen recognition via nonlinearly controlling CD8 corporation. Naturally occurring TCRs have high specificity to cognate antigens, while high-affinity engineered TCRs have low specificity.
