# Supplementary material for: TCR catch bonds nonlinearly control CD8 cooperation to shape T cell specificity
Source: Cell Res. 2025 Feb 27;35(4):265–83. doi: 10.1038/s41422-025-01077-9 (PMC11958657; doi:10.1038/s41422-025-01077-9)
Supplement: Supplementary file 12 — Table S1 [file 41422_2025_1077_MOESM12_ESM.pdf]

Supplementary information, Table S1 Summary of mean 2D effective affinities ($A_c K_a$), or zero-force off-rates (k_{off}), or zero-force on-rates ($A_c k_{on}$).

**TCR–pMHC**	**$A_c K_a$ (μm^4)**	**± SEM**	**k_{off} at 0 pN (s^{-1})**	**± SEM**	**$A_c k_{on}$ (μm^{4}s^{-1})**	**± SEM**
**2C-TCR–R4-MHC**	0.0016	0.00022	3.08	0.90	0.0054	0.0023
**2C-TCR–L4-MHC**	0.000037	0.0000052	6.61	3.79	0.00028	0.00017
**m33-TCR–R4-MHC**	0.0013	0.00025	2.70	0.49	0.0034	0.00020
**m33-TCR–L4-MHC**	0.00089	0.00023	4.22	1.67	0.0031	0.00061
**m67-TCR–R4-MHC**	0.0016	0.00024	4.97	1.65	0.0072	0.0013
**m67-TCR–L4-MHC**	0.00099	0.00019	4.92	3.75	0.0036	0.0023
**MEL8-TCR–MelanA**	0.00010	0.000030	3.20	1.16	0.00035	9.61E-05
**MEL8-TCR–IMP2**	0.000028	0.0000015	11.94	4.63	0.00034	1.82E-05
