# Supplementary material for: TCR catch bonds nonlinearly control CD8 cooperation to shape T cell specificity
Source: Cell Res. 2025 Feb 27;35(4):265–83. doi: 10.1038/s41422-025-01077-9 (PMC11958657; doi:10.1038/s41422-025-01077-9)
Supplement: Supplementary file 13 — Table S2 [file 41422_2025_1077_MOESM13_ESM.pdf]

Supplementary information, Table S2 Summary of sample sizes of bond lifetime measurements under force. The measurements (N) per bond lifetime vs. force curve are summarized according to the related figures: Figs. 2, 5, 6 and S9.

Fig. panel	Interaction	N
**Fig. 2f**	2C-TCR–R4-MHC–CD8	1406
**Fig. 2f**	2C-TCR–R4-MHC	1063
**Fig. 2g**	m33-TCR–R4-MHC–CD8	483
**Fig. 2g**	m33-TCR–R4-MHC	747
**Fig. 2h**	m67-TCR–R4-MHC–CD8	603
**Fig. 2h**	m67-TCR–R4-MHC	358
**Fig. 2i**	2C-TCR–L4-MHC–CD8	1097
**Fig. 2i**	2C-TCR–L4-MHC	1482
**Fig. 2j**	m33-TCR–L4-MHC–CD8	797
**Fig. 2j**	m33-TCR–L4-MHC	610
**Fig. 2k**	m67-TCR–L4-MHC–CD8	887
**Fig. 2k**	m67-TCR–L4-MHC	542
**Fig. 5e**	2C-TCR–R4-MHC–CD8 (Ile2Ala)	579
**Fig. 6c**	MAG-IC3-TCR–MAGE-A3	349
**Fig. 6c**	MAG-IC3-TCR–MAGE-A3–CD8	363
**Fig. 6d**	MAG-IC3-TCR–Titin	534
**Fig. 6d**	MAG-IC3-TCR–Titin–CD8	463
**Fig. S10c**	MEL8-TCR–MelanA	484
**Fig. S10c**	MEL8-TCR–MelanA–CD8	307
**Fig. S10d**	MEL8-TCR–IMP2	408
**Fig. S10d**	MEL8-TCR–IMP2–CD8	450
