# Supplementary material for: TCR catch bonds nonlinearly control CD8 cooperation to shape T cell specificity
Source: Cell Res. 2025 Feb 27;35(4):265–83. doi: 10.1038/s41422-025-01077-9 (PMC11958657; doi:10.1038/s41422-025-01077-9)
Supplement: Supplementary file 14 — Table S3 [file 41422_2025_1077_MOESM14_ESM.pdf]

Supplementary information, Table S3 Summary of the values and the numbers of TCR–pMHC mean bond lifetimes along with corresponding error bars.

TCR	peptide	CD8	Force (pN)	Mean lifetime (s)	± SEM
**2C**	R4	-	2.66	0.20	0.04
			5.45	0.27	0.03
			8.32	0.30	0.03
			11.45	0.22	0.03
			14.64	0.14	0.02
			17.22	0.11	0.02
**2C**	L4	-	3.03	0.16	0.02
			5.72	0.15	0.02
			8.77	0.12	0.02
			11.59	0.10	0.01
			15.08	0.05	0.01
**m33**	R4	-	4.69	1.99	0.54
			9.16	2.98	0.33
			12.85	3.43	0.36
			17.39	2.81	0.40
			21.13	1.67	0.55
			23.22	1.06	0.47
**m33**	L4	-	5.41	1.26	0.30
			9.62	1.82	0.37
			14.26	1.04	0.36
			17.43	0.43	0.10
			20.50	0.19	0.11
**m67**	R4	-	6.57	2.53	0.95
			9.19	4.91	0.87
			13.18	5.94	1.09
			17.16	4.46	0.97
			21.15	3.19	0.86
			24.67	1.38	0.58
**m67**	L4	-	4.65	1.07	0.52
			6.69	1.89	0.53
			9.59	2.56	0.63
			12.69	1.42	0.59
			14.47	0.60	0.29
			16.73	0.34	0.17

**MAG-IC3**	MAGE-A3	-	4.86	5.73	1.69
			8.20	11.72	3.11
			10.41	7.58	1.95
			12.76	4.91	1.86
			15.74	1.84	1.24
			19.35	0.03	0.00
**MAG-IC3**	Titin	-	5.09	2.51	0.72
			9.88	5.22	1.29
			14.89	3.78	0.78
			20.41	3.41	1.32
			22.18	2.33	0.86
			23.68	1.94	0.64
**MEL8**	MelanA	-	4.38	0.20	0.06
			6.85	0.50	0.14
			9.93	0.69	0.21
			12.70	0.55	0.14
			15.24	0.35	0.07
			19.52	0.18	0.14
**MEL8**	IMP2	-	4.23	0.24	0.07
			7.46	0.38	0.09
			10.53	0.32	0.08
			12.87	0.24	0.05
			16.24	0.15	0.06
			18.27	0.06	0.02
			20.76	0.04	0.02
