# Supplementary material for: TCR catch bonds nonlinearly control CD8 cooperation to shape T cell specificity
Source: Cell Res. 2025 Feb 27;35(4):265–83. doi: 10.1038/s41422-025-01077-9 (PMC11958657; doi:10.1038/s41422-025-01077-9)
Supplement: Supplementary file 15 — Table S4 [file 41422_2025_1077_MOESM15_ESM.pdf]

Supplementary information, Table S4 Summary of the values and the numbers of TCR–pMHC–CD8 mean bond lifetimes, along with corresponding error bars.

TCR	peptide	CD8	Force (pN)	Mean lifetime (s)	± SEM
**2C**	R4	Mouse CD8	3.53	0.17	0.05
			5.47	0.32	0.07
			7.96	0.54	0.07
			10.66	0.80	0.07
			13.34	0.65	0.08
			16.79	0.46	0.08
			19.45	0.36	0.06
			22.57	0.22	0.08
**2C**	L4	Mouse CD8	3.66	0.19	0.03
			6.45	0.11	0.01
			9.95	0.10	0.01
			14.17	0.07	0.02
			15.94	0.06	0.01
			18.57	0.04	0.02
**m33**	R4	Mouse CD8	5.90	1.83	0.63
			8.20	3.98	0.97
			12.49	5.20	0.88
			17.42	4.07	0.84
			21.29	2.43	0.94
			23.82	0.59	0.33
**m33**	L4	Mouse CD8	4.65	1.35	0.63
			6.61	2.65	1.04
			9.91	3.99	0.94
			13.75	2.35	0.86
			17.38	1.24	0.48
			20.41	0.36	0.15
**m67**	R4	Mouse CD8	5.87	2.23	0.91
			8.20	4.57	0.80
			12.53	5.95	0.79
			18.33	4.02	0.81
			21.96	1.49	0.44
			24.51	0.49	0.18
**m67**	L4	Mouse	5.52	0.77	0.38

		CD8	7.75	2.65	0.76
			10.39	5.09	1.11
			13.44	3.14	0.70
			17.04	1.51	0.46
			19.61	0.42	0.26
**2C**	R4	Mouse CD8(Ile2 Ala)	3.99	0.31	0.08
			5.93	0.46	0.09
			8.84	0.54	0.10
			12.78	0.38	0.06
			16.02	0.26	0.07
			17.02	0.16	0.07
**MAG-IC3**	MAGE-A3	Human CD8	4.39	3.05	0.85
			7.58	6.03	1.81
			11.17	8.16	1.90
			15.00	3.97	1.81
			18.04	1.79	0.67
			21.96	0.04	0.03
**MAG-IC3**	Titin	Human CD8	5.48	5.51	1.40
			8.80	10.15	2.03
			12.25	7.34	1.53
			17.44	4.74	2.70
			21.18	3.42	1.16
			24.74	1.50	0.61
			27.57	0.99	0.37
**MEL8**	MelanA	Human CD8	5.28	0.42	0.15
			7.96	0.89	0.28
			10.06	1.36	0.32
			13.23	0.81	0.32
			15.85	0.43	0.14
			19.15	0.26	0.18
**MEL8**	IMP2	Human CD8	5.23	0.45	0.32
			7.11	1.03	0.48
			9.01	1.60	0.61
			11.12	0.91	0.48
			13.19	0.42	0.26
			15.62	0.30	0.11

			19.153	0.091	0.049
