# Supplementary material for: TCR catch bonds nonlinearly control CD8 cooperation to shape T cell specificity
Source: Cell Res. 2025 Feb 27;35(4):265–83. doi: 10.1038/s41422-025-01077-9 (PMC11958657; doi:10.1038/s41422-025-01077-9)
Supplement: Supplementary file 16 — Table S5 [file 41422_2025_1077_MOESM16_ESM.pdf]

Supplementary information, Table S5 Summary of the bond lifetimes of TCR–pMHC or TCR–pMHC–CD8 binding in the 2–5 pN, 9–12 pN and 16–19 pN force regimes, along with the corresponding error bars.

**Force regime**	**2–5 pN**			**9–12 pN**			**16–19 pN**		
**TCR–pMHC pairs**	**Force (pN)**	**Mean lifetime (s)**	**± SEM**	**Force (pN)**	**Mean lifetime (s)**	**± SEM**	**Force (pN)**	**Mean lifetime (s)**	**± SEM**
**2C-TCR–R4-MHC–CD8**	4.40	0.19	0.05	9.68	0.95	0.09	17.38	0.22	0.08
**2C-TCR–R4-MHC**	3.97	0.25	0.05	9.38	0.31	0.05	17.14	0.11	0.02
**2C-TCR–L4-MHC–CD8**	3.25	0.16	0.03	9.21	0.10	0.04	16.10	0.05	0.03
**2C-TCR–L4-MHC**	3.86	0.19	0.03	10.45	0.09	0.01	16.61	0.02	0.01
**m33-TCR–R4-MHC–CD8**	4.06	0.41	0.14	10.21	10.03	3.37	16.23	4.40	1.82
**m33-TCR–R4-MHC**	4.64	1.45	0.55	10.03	2.95	0.51	17.16	2.30	0.52
**m33-TCR–L4-MHC–CD8**	4.23	1.62	0.86	9.87	8.46	2.68	17.78	0.68	0.27
**m33-TCR–L4-MHC**	4.37	1.28	0.47	9.40	1.49	0.90	16.80	0.31	0.08
**m67-TCR–R4-MHC–CD8**	4.01	0.25	0.09	9.77	7.20	1.45	17.62	4.47	1.02
**m67-TCR–R4-MHC**	5.34	0.19	0.11	9.92	6.50	1.49	17.46	4.22	1.72
**m67-TCR–L4-MHC–CD8**	4.16	0.19	0.05	9.82	7.51	1.83	17.57	2.55	0.96
**m67-TCR–L4-MHC**	4.22	0.57	0.21	9.78	1.72	0.50	16.10	1.61	0.85
**2C-TCR–R4-MHC–CD8 (Ile2Ala)**	4.28	0.46	0.12	10.22	0.46	0.11	17.08	0.14	0.04
**MAG-IC3-TCR–MAGE-A3**	3.57	0.44	0.07	10.17	8.98	2.36	17.28	0.44	0.43
**MAG-IC3-TCR–MAGE-A3–CD8**	3.90	2.37	0.90	10.39	5.82	2.20	17.30	0.99	0.49
**MAG-IC3-TCR–Titin**	3.25	0.27	0.06	10.73	4.42	2.84	17.81	3.45	1.27

**MAG-IC3-TCR–Titin–CD8**	3.58	0.12	0.02	10.32	7.25	2.29	17.46	2.78	1.82
**MEL8-TCR–MelanA**	4.11	0.19	0.11	9.62	0.69	0.21	16.88	0.45	0.16
**MEL8-TCR–MelanA–CD8**	4.14	0.27	0.12	9.87	1.36	0.32	16.63	0.30	0.13
**MEL8-TCR–IMP2**	4.09	0.21	0.10	9.67	0.51	0.18	16.89	0.09	0.03
**MEL8-TCR–IMP2–CD8**	4.45	0.14	0.07	10.24	1.48	0.57	17.03	0.21	0.13
