# Supplementary material for: TCR catch bonds nonlinearly control CD8 cooperation to shape T cell specificity
Source: Cell Res. 2025 Feb 27;35(4):265–83. doi: 10.1038/s41422-025-01077-9 (PMC11958657; doi:10.1038/s41422-025-01077-9)
Supplement: Supplementary file 17 — Table S6 [file 41422_2025_1077_MOESM17_ESM.pdf]

Supplementary information, Table S6 TCR specificities (mean) and the ratios of bi-molecular TCR–pMHC 3D binding affinities, 2D binding affinities, 2D off-rates, on-rates, bi-molecular TCR–pMHC bond lifetimes, or tri-molecular TCR–pMHC–CD8 bond lifetimes under different force regimes of antigen A to antigen B.

**TCR (CD8+/-)**	**TCR specificity (mean)**	**± SEM**	**TCR–pMHC binding affinity ratios of R4 (MAGE-A3) to L4 (Titin)**							**TCR–pMHC or TCR–pMHC–CD8 bond lifetime ratios of R4 (MAGE-A3) to L4 (Titin)**					
			**3D**	$A_{\mathrm{c}}K_{\mathrm{a}}$ **(μm^4)**	**± SEM**	k_{off} **(0 pN) (s^{-1})**	**± SEM**	$A_{\mathrm{c}}k_{\mathrm{on}}$ **(μm^4 s^{-1})**	**± SEM**	**2–5 pN**	**± SEM**	**9–12 pN**	**± SEM**	**16–19 pN**	**± SEM**
**2C**	1.07	0.071	1.89	43.59	6.01	0.47	0.24	19.49	8.28	1.30	0.30	3.38	0.66	5.10	1.67
**m33**	7820.75	7210.67	3.04	1.50	0.27	0.64	0.11	1.17	0.74	1.13	0.60	1.99	1.26	7.55	2.58
**m67**	19.93	10.49	3.66	1.63	0.24	1.01	0.34	2.12	0.38	0.34	0.23	3.77	1.40	2.63	1.75
**2C-TCR–CD8**	65460.21	37668.65								1.22	0.38	10.04	4.12	4.35	3.10
**m33-TCR–CD8**	0.0044	0.0046								0.25	0.16	1.19	0.55	6.47	3.70
**m67-TCR–CD8**	0.00083	0.00091								1.30	0.55	0.96	0.30	1.75	0.77
**2C-TCR–CD8 (Fig. 5g)**	28183.83	23775.75													
**2C-TCR–CD8 (Ile2Ala)**	7074.20	5803.08								2.89	0.97	4.81	2.26	2.75	1.85
**2C (Fig. 5g)**	1.06	0.94													
**MAG-IC3-TCR**										1.65	0.45	2.03	1.41	0.13	0.13
**MAG-IC3-TCR–CD8**										19.08	7.85	0.80	0.40	0.35	0.29
