# Supplementary material for: TCR catch bonds nonlinearly control CD8 cooperation to shape T cell specificity
Source: Cell Res. 2025 Feb 27;35(4):265–83. doi: 10.1038/s41422-025-01077-9 (PMC11958657; doi:10.1038/s41422-025-01077-9)
Supplement: Supplementary file 18 — Table S7 [file 41422_2025_1077_MOESM18_ESM.pdf]

Supplementary information, Table S7 The Pearson correlation coefficients for TCR specificities with the ratios of bi-molecular TCR–pMHC 3D binding affinities, 2D binding affinities, 2D off-rates, 2D on-rates, bi-molecular TCR–pMHC bond lifetimes, or tri-molecular TCR–pMHC–CD8 bond lifetimes under different force regimes of R4 to L4.

	**The Pearson correlation coefficients (*r*) for TCR specificities with different kinetic metrics**						
**Metrics** / **Conditions**	3D	2D					
	K_a	$A_c K_a$	k_{off} at 0 pN	$A_c k_{on}$	2–5 pN	9–12 pN	16–19 pN
**CD8-**	0.17	−0.50	−0.20	−0.54	0.35	−0.98	−0.22
**CD8+**	N.D.	N.D.	N.D.	N.D.	0.44	0.99	0.059
