# Supplementary material for: TCR catch bonds nonlinearly control CD8 cooperation to shape T cell specificity
Source: Cell Res. 2025 Feb 27;35(4):265–83. doi: 10.1038/s41422-025-01077-9 (PMC11958657; doi:10.1038/s41422-025-01077-9)
Supplement: Supplementary file 19 — Table S8 [file 41422_2025_1077_MOESM19_ESM.pdf]

Supplementary information, Table S8 Summary of the CD8 enhancement power to TCR–pMHC bi-molecular lifetimes.

Forge regime	2–5 pN		9–12 pN		16–19 pN	
Metrics / TCR-pMHC pairs	CD8 enhancement power	± SEM	CD8 enhancement power	± SEM	CD8 enhancement power	± SEM
**2C-TCR–L4-MHC**	0.83	0.21	1.02	0.42	2.43	1.63
**m33-TCR–L4-MHC**	1.26	0.81	5.70	3.90	2.23	1.05
**m67-TCR–L4-MHC**	0.34	0.15	4.36	1.65	1.59	1.03
**2C-TCR–R4-MHC**	0.78	0.23	3.04	0.57	2.07	0.83
**m33-TCR–R4-MHC**	0.28	0.14	3.40	1.28	1.91	0.90
**m67-TCR–R4-MHC**	1.31	0.86	1.11	0.34	1.06	0.49
**2C-TCR–R4-MHC–CD8 (Ile2Ala)**	1.85	0.59	1.46	0.43	1.31	0.44
**MAG-IC3-TCR–MAGE-A3**	5.40	2.21	0.65	0.30	2.25	2.45
**MAG-IC3-TCR–Titin**	0.47	0.13	1.64	1.18	0.81	0.61
