# Supplementary material for: TCR catch bonds nonlinearly control CD8 cooperation to shape T cell specificity
Source: Cell Res. 2025 Feb 27;35(4):265–83. doi: 10.1038/s41422-025-01077-9 (PMC11958657; doi:10.1038/s41422-025-01077-9)
Supplement: Supplementary file 7 — Fig. S7 [file 41422_2025_1077_MOESM7_ESM.pdf]

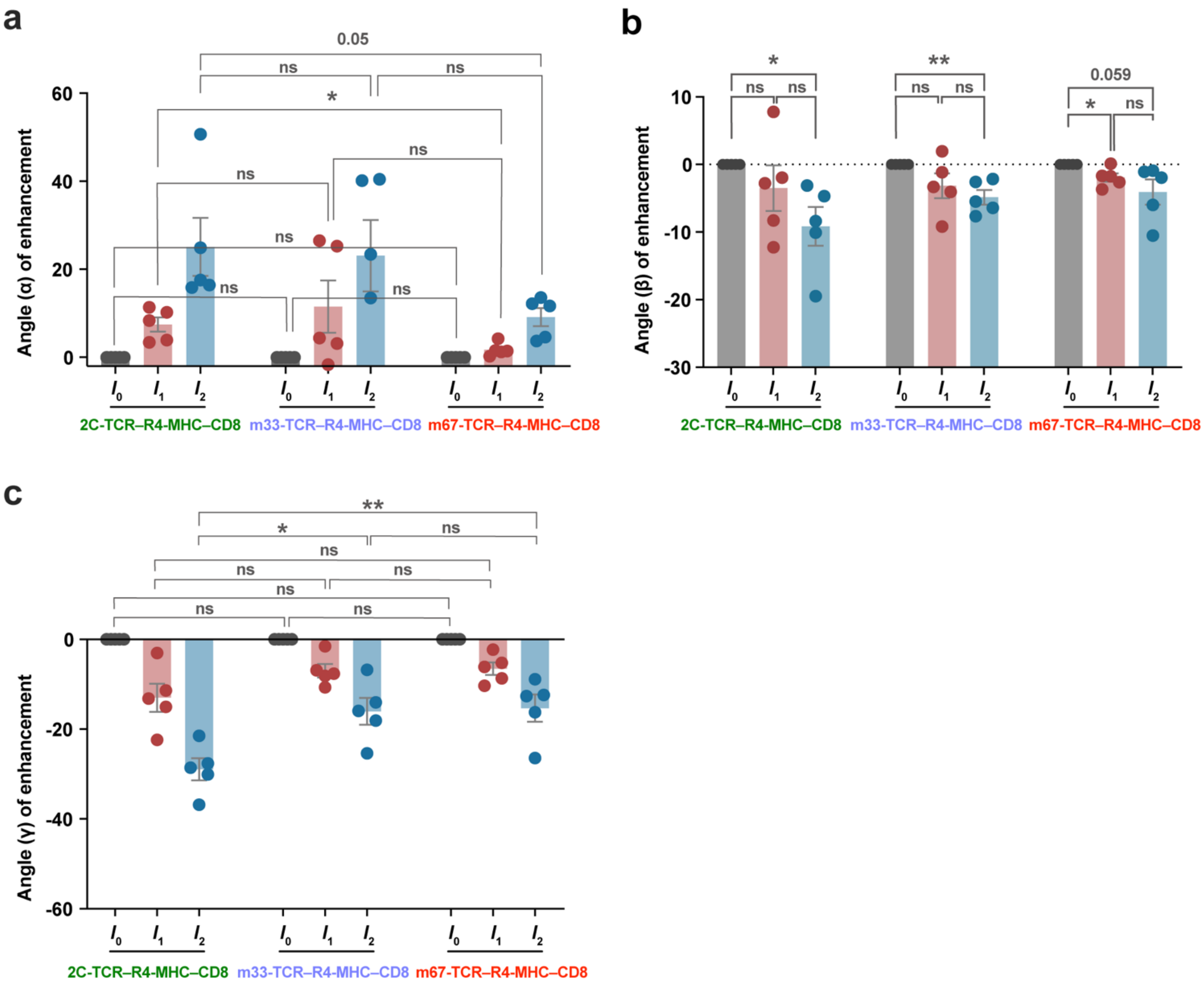

Supplementary information, Fig. S7 Force-induced angle changes in cv-SMD simulations.

a–**c** Force-induced angle changes of α (**a**), β (**b**) and γ (**c**) for the 2C-, or m33-, or m67-TCRs in the presence of CD8 binding with R4-MHC in cv-SMD simulations. The data for 2C- and m67-TCRs from Fig. 4c, d was replotted in Fig. S7a, c for comparison. Error bars are ± SEMs. The statistical analyses were performed by unpaired *t*-tests; the statistical significance was indicated as follows: **P* < 0.05, ***P* < 0.01, ****P* < 0.005, *****P* < 0.0001.
